# An Integrated Analysis Reveals Geniposide Extracted From *Gardenia jasminoides J.Ellis* Regulates Calcium Signaling Pathway Essential for Influenza A Virus Replication

**DOI:** 10.3389/fphar.2021.755796

**Published:** 2021-11-19

**Authors:** Lirun Zhou, Lei Bao, Yaxin Wang, Mengping Chen, Yingying Zhang, Zihan Geng, Ronghua Zhao, Jing Sun, Yanyan Bao, Yujing Shi, Rongmei Yao, Shanshan Guo, Xiaolan Cui

**Affiliations:** ^1^ Institute of Chinese Materia Medica, China Academy of Chinese Medical Sciences, Beijing, China; ^2^ Dongzhimen Hospital, Beijing University of Chinese Medicine, Beijing, China

**Keywords:** geniposide, influenza A virus, RNA polymerase, calcium signaling pathway, CAMKII

## Abstract

Geniposide, an iridoid glycoside purified from the fruit of *Gardenia jasminoides J.Ellis*, has been reported to possess pleiotropic activity against different diseases. In particular, geniposide possesses a variety of biological activities and exerts good therapeutic effects in the treatment of several strains of the influenza virus. However, the molecular mechanism for the therapeutic effect has not been well defined. This study aimed to investigate the mechanism of geniposide on influenza A virus (IAV). The potential targets and signaling pathways of geniposide in the IAV infection were predicted using network pharmacology analysis. According to the result of network pharmacology analysis, we validated the calcium signaling pathway induced by IAV and investigated the effect of geniposide extracted from *Gardenia jasminoides J.Ellis* on this pathway. The primary Gene Ontology (GO) biological processes and Kyoto Encyclopedia of Genes and Genomes (KEGG) pathways KEGG enrichment analysis indicated that geniposide has a multi-target and multi-pathway inhibitory effect against influenza, and one of the mechanisms involves calcium signaling pathway. In the current study, geniposide treatment greatly decreased the levels of RNA polymerase in HEK-293T cells infected with IAV. Knocking down CAMKII in IAV-infected HEK-293T cells enhanced virus RNA (vRNA) production. Geniposide treatment increased CAMKII expression after IAV infection. Meanwhile, the CREB and c-Fos expressions were inhibited by geniposide after IAV infection. The experimental validation data showed that the geniposide was able to alleviate extracellular Ca^2+^ influx, dramatically decreased neuraminidase activity, and suppressed IAV replication *in vitro via* regulating the calcium signaling pathway. These anti-IAV effects might be related to the disrupted interplay between IAV RNA polymerase and CAMKII and the regulation of the downstream calcium signaling pathway essential for IAV replication. Taken together, the findings reveal a new facet of the mechanism by which geniposide fights IAV in a way that depends on CAMKII replication.

## Introduction

Influenza A virus (IAV) is common in all age group populations. It has been regarded as a severe public health concern. It is one of the most common infectious diseases with a high incidence rate and high mortality rate (Tregoning et al., 2018). Although there are 26 licensed inactivated vaccines that are available for the prevention of influenza, the number of the death caused by epidemics worldwide is still estimated as 300,000–650,000 every year (Palache et al., 2014; Behzadi and Leyva-Grado, 2019). As IAV rapidly evolves through antigenic drift and shift, new subtype strains continuously emerge (Zhang et al., 2019b; Jia et al., 2019). For example, the most recent influenza pandemic caused by the H1N1 pdm09 influenza virus originated from swine spread rapidly to nearly all countries and territories (Jain et al., 2009; Smith et al., 2009). Compared to seasonal influenza viruses, the H1N1 pdm09 influenza virus causes more severe disease and deaths among adults aged 18–64 years (Writing Committee of the WHO Consultation on Clinical Aspects of Pandemic Influenza, 2010; Chiu et al., 2011). Human infection with H7N9 influenza viruses of avian origin emerged in March 2013 in China, and these viruses have continued to spread into populations with unprecedented mortality and morbidity (Zhu et al., 2016; Su et al., 2017). Obviously, influenza has the potential for mortality, high mutation rates, and pandemic risk; thus, it is crucial to learn more about the virulence and pathogenicity of influenza and identify the targets for the development of new drugs (Generous et al., 2014).

Antiviral drugs play a critical role in preventing influenza epidemics and pandemics, especially for antigenically different strains or new subtypes (Enkirch et al., 2019). Currently, the anti-influenza drugs approved by the Federal Drug Administration (FDA) can be divided into two classes: adamantane-based M2 ion channel blockers, which inhibit viral replication by preventing endosome acidification and viral ribonucleoprotein delivery into the cytoplasm, and neuraminidase (NA) inhibitors, which inhibit the release of newly formed virus particles from infected cells (Doll et al., 2017; Ison, 2017). However, surveillance research reported almost all circulating human IAVs are adamantine-resistant, and the virus’ resistance to NA inhibitors emerged rapidly after the widespread application (Deyde et al., 2007; Gubareva et al., 2017). Given the inherent limitations of drugs targeting viral proteins, repurposed novel drugs targeting cellular may be a promising complement (de Chassey et al., 2014).

Host cellular proteins and pathways are involved in the influenza virus life cycle (Karlas et al., 2010; Ackerman et al., 2018). The outcomes of influenza pathogenesis are dependent on the interaction between the virus and the host cellular protein and the activation of signal transduction pathways (Dai et al., 2011; Zhao et al., 2017). The host cellular proteins and signaling pathways may facilitate virus replication by the hijacking of host molecular machinery required for the viral life cycle or trigger host innate immune defense to inhibit the virus (Zhao et al., 2017). Ca^2+^ influx may play a crucial role in the regulation of influenza A entry and infection. It has been demonstrated that influenza A infection induces Ca^2+^ oscillations of host cells, and the infection is obviously attenuated by Ca^2+^ chelation (Fujioka et al., 2013). As current examples showed that in the early stage of infection, viral glycoprotein hemagglutinin (HA) binds to voltage-dependent Ca^2+^ channel on the host cell surface to induce intracellular Ca^2+^ oscillations and mediate IAV entry, and subsequently evoke host cell calcium signaling pathway and facilitate infection (Fujioka et al., 2018).

*Gardenia jasminoides J.Ellis* (Rubiaceae), called Zhi-Zi in the Chinese pharmacopoeias, is an important heat-clearing and detoxifying Chinese herb (Wang et al., 2018). It has been used for the treatment of inflammation, acute febrile disease, ischemia/reperfusion injury, and hepatic disorders for a long history in East Asia (Lee et al., 2014; Oliveira et al., 2017; Rong et al., 2017; Li et al., 2019a). Geniposide, a type of iridoid glycoside, is the main bio-active component isolated from *Gardenia jasminoides J.Ellis* (Zhou et al., 2019). Geniposide has diverse pharmacological activities, including antioxidant, anti-inflammatory, neuroprotective, and antithrombotic actions (Zhang et al., 2013; Zhang et al., 2019a; Li et al., 2019b; Zhang et al., 2019c). Moreover, accumulated pieces of evidence have verified the antiviral properties of Geniposide, which inhibited the infection of influenza A (H1N1) pdm09 virus, enterovirus 71 virus, and Epstein–Barr virus *in vitro* and *in vivo* (Lin et al., 2013; Son et al., 2015; Zhang et al., 2017). Indeed, our previous study showed that iridoid glycoside extracted from *Fructus Gardeniae* inhibited extracellular Ca^2+^ influx induced by IAV, but the precise mechanisms need to be disclosed (Guo et al., 2014).

In the current study, in order to better understand the intervention mechanism of geniposide for influenza virus, we first constructed the PPI network between influenza virus-related molecules, and geniposide-related genes were mapped to the network to discover the correlation between them. Then, after module identification, the primary GO biological processes and KEGG pathways were identified by enrichment analysis. According to the results of the network, we validate the calcium signaling pathway induced by the IAV and investigate the effect of geniposide extracted from *Gardenia jasminoides J.Ellis* on it. The NA activity of IAV and extracellular Ca^2+^ influx were detected in MDCK cells, protein expressions in calcium signaling (CAMK II, CREB, and c-fos) were evaluated in A549 cells, and the RNA polymerase activity of influenza virus was determined in HEK-293T cells transfected with CAMK II siRNA.

## Materials and Methods

### Biosafety Statement

All experiments involved with live IAV were carried out in the Animal Biosafety Level 2 Laboratory (ABSL-2) in the Institute of Chinese Materia Medica, China Academy of Chinese Medical Sciences.

### Cells and Virus Stock

MDCK, A549, and HEK-293T cells were provided by Cell Center, Institute of Basic Medical Sciences, Peking Union Medical College (Beijing, China) and cultured in Dulbecco’s modified Eagle’s medium (DMEM) (Gibco) containing 10% heat-inactivated FBS (Gibco). For the validation studies, A FM/1/47 (H1N1) influenza virus (ATCC-VR-1754-ATC) was used. The virus was propagated in 10-day-old embryonated chicken eggs (Merial Vital Laboratory Animal Technology Co., Ltd., Beijing, China) according to the method previously described (Spackman and Killian, 2014). Virus was titrated in MDCK cells and titers (median tissue culture infective dose, TCID50) calculated to be 10^−4.5^ by the Reed–Muench method (Spackman et al., 2019).

### Sample of Geniposide Extracted From *Gardenia jasminoides J.Ellis*

Geniposide was extracted from *Gardenia jasminoides J.Ellis* with 70% ethanol, and the purity was 39.4% with the purity of iridoid glycosides >90% detected by UV spectrophotometry.

### Acquisition of Related Gene of Influenza Virus and Geniposide

Genes related to the influenza virus were searched in the OMIM (https://www.ncbi.nlm.nih.gov/omim/) database. Enter geniposide into STITCH (http://stitch.embl.de/), GeneCards (https://www.genecards.org/), and CTD (http://ctdbase.org/) database to search for related genes.

### Network Construction and Module Identification

Influenza virus-related molecules were based on the STRING (Version 9.05) database, and the virus-related molecules and first-order neighbor protein interaction network were constructed with *Homo sapiens* as the background. Geniposide-related genes were mapped to this network to understand the correlation between them. We used MCODE (http://baderlab.org/Software/MCODE), a clustering algorithm-based software, to identify gene network modules and modularity of each network (Parameters: Degree Cutoff = 2, Node Score Cutoff = 0.2, Max Depth = 100, K-core threshold = 2).

### Functional Enrichment Analysis

In this study, DAVID 6.7 (http://david.abcc.ncifcrf.gov/) software was used for functional enrichment analysis of each module, with species restricted to *Homo sapiens*. As primary GO (Gene Ontology) biological process and KEGG (Kyoto Encyclopedia of Genes and Genomes) pathway can describe the biological features of modules. Modified Fisher’s exact test and Benjamini were utilized for calculating and correcting *p*-value (*p* < 0.05).

### Neuraminidase Activity of Influenza A Virus

The antiviral activity against IAV of geniposide was elevated by NA activity assay. MDCK cells were seeded in 96-well plates at a density of 1 × 10^5^ cells/ml and infected with the FM/1/47 strain of H1N1 influenza virus (100TCID50, 100 μl/well) for 1 h. Then, the infected cells were treated with serially diluted geniposide solutions with 320, 160, 80, and 40 μg/ml, respectively. Ribavirin served as a reference drug, at a final concentration of 3.13 mg/ml. After incubation at 37°C for 48 h, the cells were harvested and NA activity was determined by NA assay kit (Beyotime, Shanghai, China) according to the manufacturer’s protocol.

### Confocal Microscopy

MDCK cells were cultured on 35-mm glass-bottom culture dishes (NEST, China) at a concentration of 5 × 10^5^ cells/ml. When the cells grew to 80%–90% confluence, influenza virus A/FM1/47 solution (100TCID50) was added to the cultures. After incubation at 37°C for 30 min, MDCK cells were loaded with 10 mg/ml of fluo-3/AM probe (Invitrogen) for 30 min to detect the intracellular calcium concentration, and then photographed by a laser confocal microscope (Olympus, FV1000, Japan). For the measurement of Ca^2+^ influx, the samples were excited by an argon laser at 488 nm, and the fluorescence intensity of emission was detected at the 530-nm wavelength.

### Western Blotting Assay

Post-infection of influenza virus A/FM1/47 at 12, 24, 36, and 48 h, A549 cells were lysed in RIPA lysate buffer that was supplemented with PMSF and protease inhibitor cocktail (Sigma). The determination of total protein concentrations was performed by Bicinchoninic Acid (BCA) kit to ensure equal sample loading. Proteins were separated on 10% SDS-PAGE gel and then transferred onto a 0.45-μm NC membrane. After blocking with non-fat milk, the blots were incubated with the primary antibodies: CAMKII and GAPDH (1:2,000, Abcam, United States) and CREB and c-fos (1:1,500, CST, United States), overnight at 4°C. Subsequently, the secondary antibody incubation was conducted with goat anti-rabbit IgG antibody (1:20,000) for 3 h at room temperature. Blots were visualized by ECL and the density of bands was determined by ImageJ software.

### Dual-Luciferase Reporter Gene Assay

HEK-293T cells were transfected with CAMKII siRNA (forward primer 5′-CAC​CAC​CAU​UGA​GGA​GGA​ATT-3′ and reverse primer 5′-UUC​CUC​CUC​AAU​GGU​GGU​GTT-3′) at 37°C, 5% CO_2_. After 12 h, the cells were co-transfected with four plasmids containing the cDNA of RdRP (RNA-dependent RNA polymerase) of influenza virus A/WSN/33(H1N1) (pHW181-PB2, pHW182-PB1, pHW183-PA, pHW185-NP) or negative control (pFlu-luc) using Lipofectamine 3000 transfection kit (Invitrogen, United States) according to the previous study (Wang et al., 2012). After 12 h of transfection, the supernatant was discarded and cells were treated with different concentrations of geniposide (320 and 160 μg/ml) for 24 h at 37°C, 5% CO_2_. Luciferase activity was finally detected by a dual-luciferase reporter assay system (Promega, United States) according to the manufacturer’s instructions. The relative luciferase activities were determined by the ratio of Renilla luciferase value to firefly luciferase value.

### Statistical Analysis

Experimental data were presented as mean ± standard error of the mean. Differences were analyzed by GraphPad Prism 7.0 software system with one-way analysis of variance (ANOVA), and significant differences were determined by the Bonferroni test. Differences were considered to be significant if *p* values < 0.05.

## Results

### Related Gene of Influenza Virus and Geniposide

Based on the OMIM database, a total of 77 genes related to influenza viruses were obtained. A total of 35 genes related to geniposide were searched from databases such as STITCH, GeneCards, and CTD.

### Network and Module Identification

**FIGURE 1:**
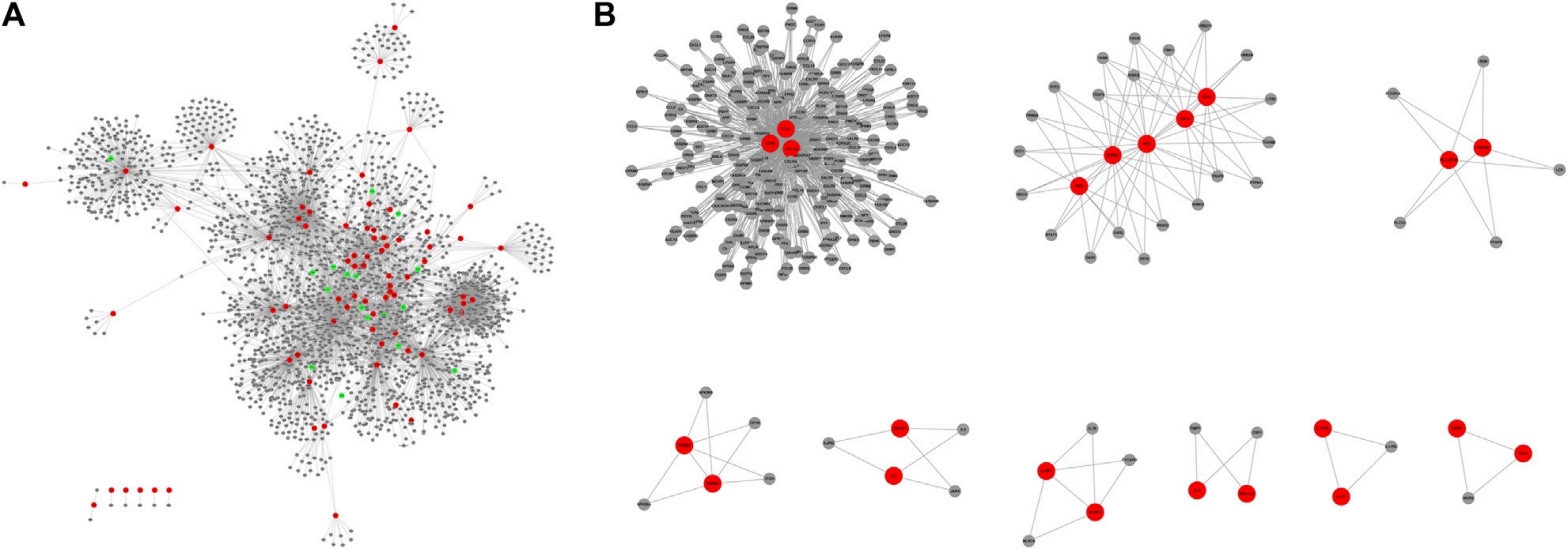
In the figure, the red node represents the influenza virus-related molecule, the gray node represents the first-order neighbor of the influenza virus-related molecule, and the green represents the geniposide-related molecule.

The PPI network constructed from virus-related molecules and first-order neighbor consists of 2,395 nodes and 5,574 edges (score ≥0.9). These geniposide-related genes were mapped to PPI networks, and 17 were found to be associated with influenza virus networks (Figure 1A). After the MCODE method, there were nine modules that consist of more than three nodes from the PPI network, sorted according to the score (Figure 1B; Table 1).

**TABLE 1:** The modules from network.

Module	Score	Node	Edge	Name of nodes
1	2.973	223	663	TAS2R60, CCR7, CCL4, CXCL8, CXCR5, CXCL10, CCR1, CCR3, CXCL9, PF4, CCR2, CXCL1, CXCL12, CXCR3, CXCR4, CXCL11, CXCL2, CCL4L1, CCL13, CCL20, CCL27, CXCL5, CX3CL1, PPBP, CCL25, CCR4, CXCR2, CXCL3, CCL1, CXCR1, CXCL6, CX3CR1, CCR10, CXCL16, CXCR6, C3AR1, CCR9, CCR8, CCL16, GNAI2, APLN, ANXA1, APP, GPR183, AGT, AGTR2, ACKR3, S1PR1, C5AR1, GNAI1, GNAI3, CCL28, FPR3, ADORA3, SAA1, FPR2, C3, S1PR3, CASR, FPR1, S1PR2, GNAT3, OPRM1, PTGDR2, S1PR4, KNG1, P2RY12, GNGT2, CNR1, S1PR5, SST, C5, OPRL1, POMC, P2RY13, APLNR, CNR2, NPY, GNB3, CORT, NPY1R, NPY2R, DRD2, DRD3, OPRK1, HCAR2, PTGER3, HRH4, GPR18, ADORA1, OPRD1, GABBR1, GRM8, GNB4, GNG11, GNB2, GNB5, GNG4, GNG2, TAS1R3, GNB1, GRM2, GNG7, P2RY14, PNOC, GNG10, GPR31, GNG5, HCAR3, CHRM2, GNG12, GNGT1, GNG8, GNG13, GNG3, PMCH, GRM6, GPSM3, GRM3, ADCY4, SUCNR1, LPAR5, LPAR2, GRM4, GPR55, HCAR1, PENK, HTR1B, ADCY7, ADRA2A, LPAR3, DRD4, OXER1, GRM7, GABBR2, PYY, LPAR1, BDKRB1, TAS2R7, ADRA2C, GAL, ADRA2B, GPER1, PDYN, ADCY8, ADCY3, SSTR3, HRH3, ADCY5, ADCY1, ADCY6, ADCY2, NMUR1, OXGR1, GPR17, ADCY9, HTR1D, PSAP, NPBWR1, BDKRB2, MCHR1, MCHR2, HTR1A, CHRM4, TAS2R39, TAS1R1, TAS2R41, TAS2R38, GPR37, TAS2R46, NPY5R, NPY4R, NPBWR2, NMUR2, P2RY4, PCP2, SSTR5, SSTR2, SSTR4, HTR1F, MTNR1A, GALR2, TAS2R31, TAS2R1, TAS1R2, TAS2R16, HTR5A, TAS2R14, HTR1E, GPSM2, NMS, NMU, TAS2R42, TAS2R19, TAS2R10, TAS2R30, RXFP4, TAS2R40, INSL5, MTNR1B, TAS2R50, RXFP3, TAS2R4, TAS2R3, SSTR1, TAS2R5, GALR3, TAS2R20, TAS2R13, TAS2R9, NPB, GPR37L1, PPY, HEBP1, TAS2R8, GALR1, GPSM1, TAS2R43, CCR5, NPW, CCL5, RLN3, CCL19
2	2.923	26	76	IKBKE, OASL, ISG15, IFIT1, OAS1, RSAD2, STAT1, TRIM25, IFIT3, MX2, IFIT2, LY96, TRAF6, TRAF3, TBK1, IKBKG, PTPN11, UBE2N, TICAM2, TLR4, TANK, DDX58, CHUK, IRF7, UBE2V1, CD14
3	1.5	6	9	CFTR, PSMB9, NFKBIB, ITCH, NFKBIA, PSME3
4	1.429	7	10	LCK, B2M, PTAFR, FCGR1A, PIK3R2, PLCG1, HLA-DPA1
5	1.4	5	7	PYCARD, NLRC4, NLRP3, IL1B, CASP1
6	1.2	5	6	JAK3, IL7, IL2RG, IL2, MAPK1
7	1	3	3	IL17RC, IL17RA, IL17F
8	1	4	4	CSF1, IL10, TIMP1, DNAJC3
9	1	3	3	NCR2, NCR1, NCR3

### Functional Enrichment Analysis of Modules

Based on DAVID 6.7 (http://david.abcc.ncifcrf.gov/) software, there were 14 pathways (*p* < 0.05) in Module 1: Chemokine signaling pathway, Neuroactive ligand–receptor interaction, Taste transduction, Cytokine–cytokine receptor interaction, Gap junction, Melanogenesis, Progesterone-mediated oocyte maturation, Intestinal immune network for IgA production, Dilated cardiomyopathy, GnRH signaling pathway, Complement and coagulation cascades, Oocyte meiosis, Vascular smooth muscle contraction, and Calcium signaling pathway (Table 2). There were 11 pathways (*p* < 0.05) in Module 2: Toll-like receptor signaling pathway, RIG-I-like receptor signaling pathway, Cytosolic DNA-sensing pathway, Small cell lung cancer, Pathogenic *Escherichia coli* infection, NOD-like receptor signaling pathway, Adipocytokine signaling pathway, Pathways in cancer, Epithelial cell signaling in *Helicobacter pylori* infection, Pancreatic cancer, and Chronic myeloid leukemia (Table 3). There was 1 pathway (*p* < 0.05) in Module 3: Proteasome (Table 4).

**TABLE 2:** The pathway of module 1.

Pathway	Gene	*p*-value
Chemokine signaling pathway	ADCY3, ADCY4, ADCY1, ADCY2, ADCY7, ADCY8, ADCY5, ADCY6, CXCR1, CXCR2, CXCR3, CXCL11, CXCL12, CXCL10, GNG8, CXCR5, CXCR4, CCR10, CXCR6, GNG2, GNG3, GNG4, GNG5, GNG7, CCL4L1, CCR9, CCR8, CCR7, PPBP, CCR5, GNB2, CCR4, GNB1, CCR3, CCR2, CX3CR1, GNB5, GNB4, GNB3, CXCL1, CCL1, GNAI3, CXCL5, GNAI2, GNAI1, CCR1, CXCL3, CXCL2, CXCL9, GNG13, GNG11, PF4, CXCL6, CX3CL1, GNG12, CCL5, CCL28, CCL4, CCL27, CCL25, CCL20, CCL19, CCL16, GNGT1, GNGT2, CCL13, ADCY9, CXCL16, GNG10	1.657958766912765E-55
Neuroactive ligand–receptor interaction	OPRM1, MCHR1, MCHR2, ADORA3, GABBR1, LPAR3, LPAR2, GABBR2, LPAR1, ADORA1, S1PR2, S1PR3, AGTR2, HTR1B, HTR1A, S1PR1, GALR1, NMUR1, NMUR2, GALR3, S1PR4, GALR2, S1PR5, HTR1D, HTR1F, HTR5A, HTR1E, PTGER3, C5AR1, NPBWR1, NPBWR2, SSTR4, SSTR5, GRM4, GRM3, SSTR2, GRM2, SSTR3, CHRM4, SSTR1, GRM8, CHRM2, GRM7, GRM6, C3AR1, DRD3, DRD2, OPRK1, NPY2R, DRD4, FPR1, FPR3, BDKRB1, FPR2, BDKRB2, APLNR, HRH3, P2RY4, CNR1, CNR2, HRH4, ADRA2A, ADRA2C, ADRA2B, OPRL1, NPY1R, NPY5R, P2RY13, P2RY14, MTNR1B, MTNR1A, OPRD1	9.657764704734148E-49
Taste transduction	ADCY4, TAS2R1, TAS2R4, ADCY8, TAS2R5, ADCY6, TAS2R3, GNG13, TAS1R3, TAS1R1, TAS1R2, TAS2R60, TAS2R46, TAS2R9, TAS2R42, TAS2R43, TAS2R8, TAS2R7, GNG3, TAS2R20, TAS2R40, TAS2R41, GNAT3, TAS2R16, GRM4, TAS2R13, TAS2R39, TAS2R50, TAS2R14, TAS2R19, GNB1, TAS2R38, TAS2R31, GNB3, TAS2R10	2.0267715150108459E-38
Cytokine–cytokine receptor interaction	CXCL1, CCL1, CXCL5, CXCL3, CCR1, CXCL2, CXCL9, CXCR1, PF4, CXCR2, CXCL6, CXCR3, CX3CL1, CCL5, CXCL11, CCL4, CXCL12, CCL28, CCL27, CXCL10, CCL25, CXCR5, CCL20, CXCR4, CXCR6, CCR10, CCL4L1, CCL19, CCL16, CCR9, CCR8, CCL13, CCR7, PPBP, CCR5, CCR4, CCR3, CXCL16, CCR2, CX3CR1	1.4758873776336994E-15
Gap junction	ADCY3, ADCY4, ADCY1, ADCY2, GNAI3, ADCY7, GNAI2, DRD2, ADCY8, GNAI1, ADCY5, ADCY6, LPAR1, ADCY9	9.270005860103292E-6
Melanogenesis	ADCY3, ADCY4, ADCY1, ADCY2, GNAI3, GNAI2, ADCY7, ADCY8, GNAI1, ADCY5, ADCY6, POMC, ADCY9	1.356035255661388E-4
Progesterone-mediated oocyte maturation	ADCY3, ADCY4, ADCY1, ADCY2, GNAI3, ADCY7, GNAI2, ADCY9, GNAI1, ADCY8, ADCY5, ADCY6	1.5948731269833238E-4
Intestinal immune network for IgA production	CCR9, CCL25, CXCR4, CCR10, CCL28, CXCL12, CCL27	0.006412361067992899
Dilated cardiomyopathy	ADCY3, ADCY4, ADCY1, ADCY2, ADCY7, ADCY9, ADCY8, ADCY5, ADCY6	0.013743836429085802
GnRH signaling pathway	ADCY3, ADCY4, ADCY1, ADCY2, ADCY7, ADCY9, ADCY8, ADCY5, ADCY6	0.019520336471638235
Complement and coagulation cascades	KNG1, C3AR1, C5AR1, C3, C5, BDKRB1, BDKRB2	0.031244059596336946
Oocyte meiosis	ADCY3, ADCY4, ADCY1, ADCY2, ADCY7, ADCY9, ADCY8, ADCY5, ADCY6	0.03592138311782549
Vascular smooth muscle contraction	ADCY3, ADCY4, ADCY1, ADCY2, ADCY7, ADCY9, ADCY8, ADCY5, ADCY6	0.039354698598733834
Calcium signaling pathway	ADCY3, ADCY4, ADCY1, ADCY2, PTGER3, ADCY7, ADCY9, ADCY8, CHRM2, BDKRB1, BDKRB2, HTR5A	0.040643127061523385

**TABLE 3:** The pathway of module 2.

Pathway	Gene	*p*-value
Toll-like receptor signaling pathway	IKBKE, TBK1, LY96, IRF7, IKBKG, TICAM2, TLR4, TRAF6, STAT1, CHUK, CD14, TRAF3	1.2128632952757494E-15
RIG-I-like receptor signaling pathway	DDX58, IKBKE, ISG15, TBK1, IRF7, IKBKG, TRIM25, TRAF6, CHUK, TANK, TRAF3	2.6132796236858782E-15
Cytosolic DNA-sensing pathway	DDX58, IKBKE, TBK1, IRF7, IKBKG, CHUK	6.896843891896822E-7
Small cell lung cancer	IKBKG, TRAF6, CHUK, TRAF3	0.0025018429161348032
Pathogenic *Escherichia coli* infection	LY96, TLR4, CD14	0.015072927052996406
NOD-like receptor signaling pathway	IKBKG, TRAF6, CHUK	0.017685026048156277
Adipocytokine signaling pathway	IKBKG, CHUK, PTPN11	0.020476455840164474
Pathways in cancer	IKBKG, TRAF6, STAT1, CHUK, TRAF3	0.020691625131975113
Epithelial cell signaling in *Helicobacter pylori* infection	IKBKG, CHUK, PTPN11	0.021055736236424673
Pancreatic cancer	IKBKG, STAT1, CHUK	0.023441273355369472
Chronic myeloid leukemia	IKBKG, CHUK, PTPN11	0.02530096564417107

**TABLE 4:** The pathway of module 3.

Pathway	Gene	*p*-value
Proteasome	PSME3, PSMB9	0.045385433295782915

**FIGURE 2:**
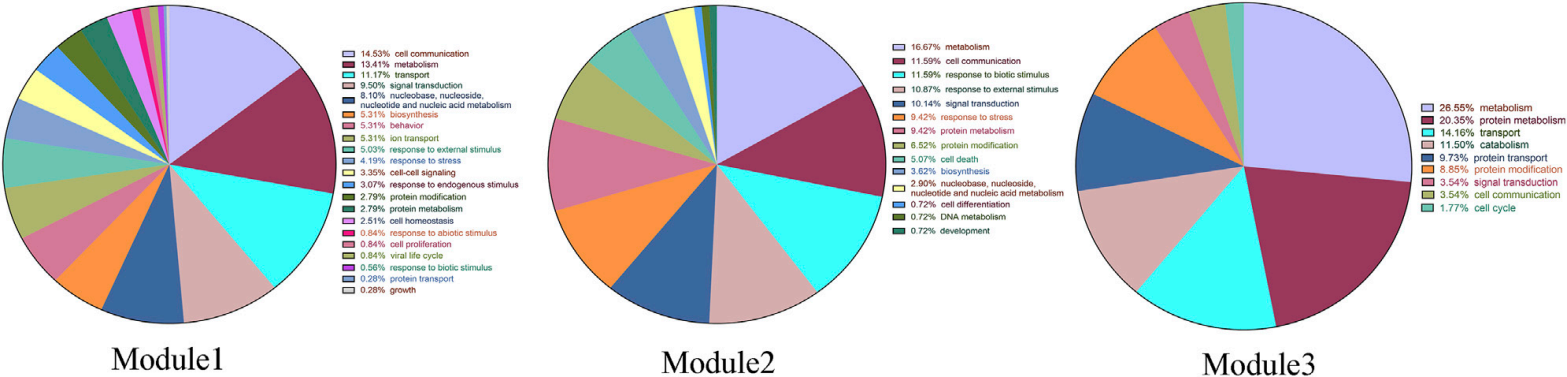
The GO-BP classification chart of three modules.

Module 1 is enriched with 326 GO biological processes, with 21 biological functional annotations, including 52 for cell communication; 48 for metabolism; 40 for transport; 34 for signal transduction; 29 for nucleobase, nucleoside, nucleotide, and nucleic acid metabolism; 19 for biosynthesis; 19 for behavior; 19 for ion transport; 18 for response to external stimulus; 15 for response to stress; 12 for cell–cell signaling; 11 for response to endogenous stimulus; and 10 for protein modification (*p* < 0.05). Module 2 is enriched with 113 GO biological processes (*p* < 0.05). Module 3 is enriched with 64 GO biological processes (*p* < 0.05) (Figure 2).

**FIGURE 3:**
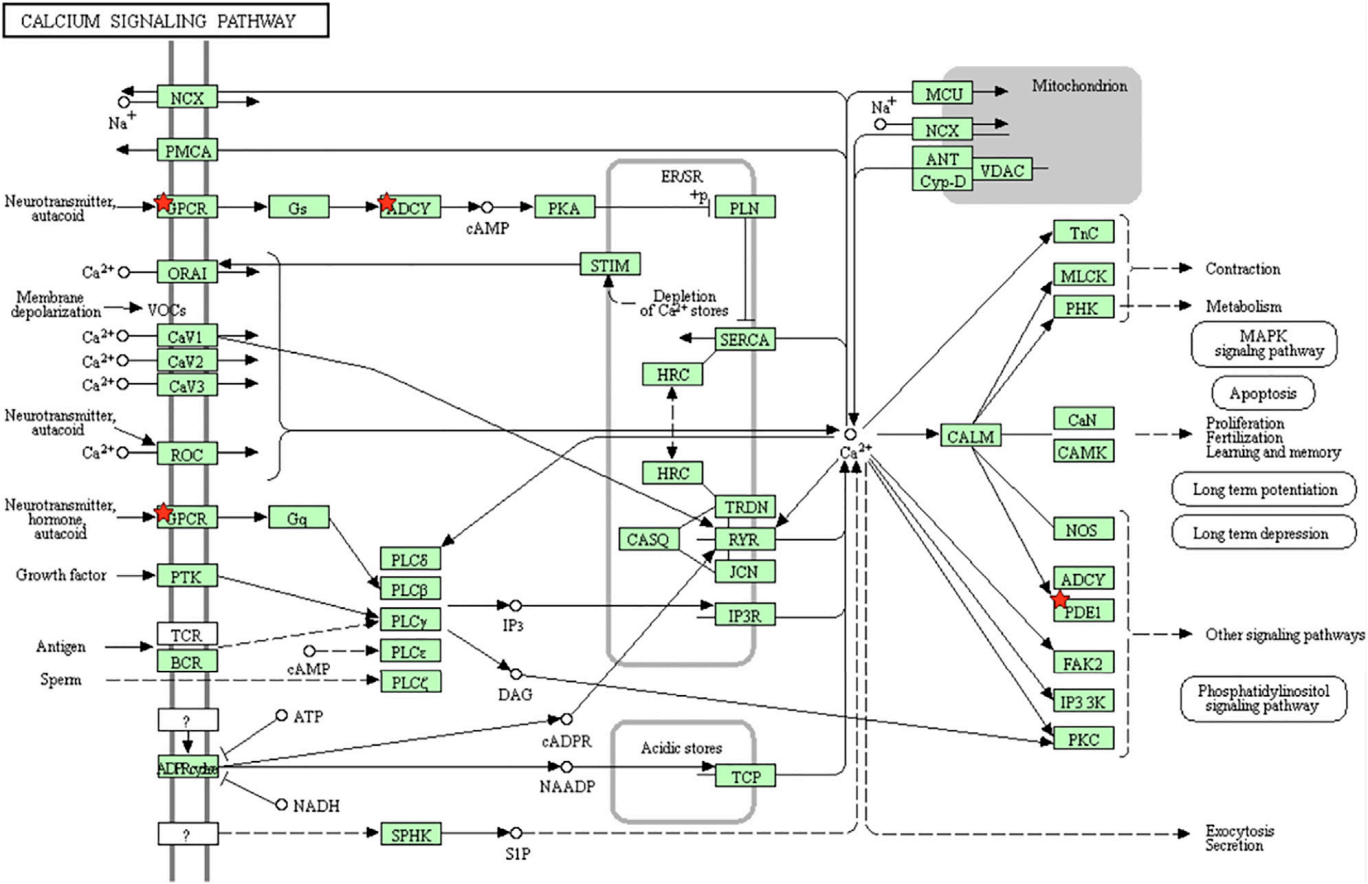
Overview of the Calcium signaling pathway. The red stars in the figure are the molecules in the module that hit the signaling pathway.

Furthermore, to verify the results from GO analysis, the genes of the Calcium signaling pathway in module 2 were enriched by KEGG pathway analysis (Figure 3).

### Geniposide Inhibits Neuraminidase Activity of Influenza A Virus

**FIGURE 4:**
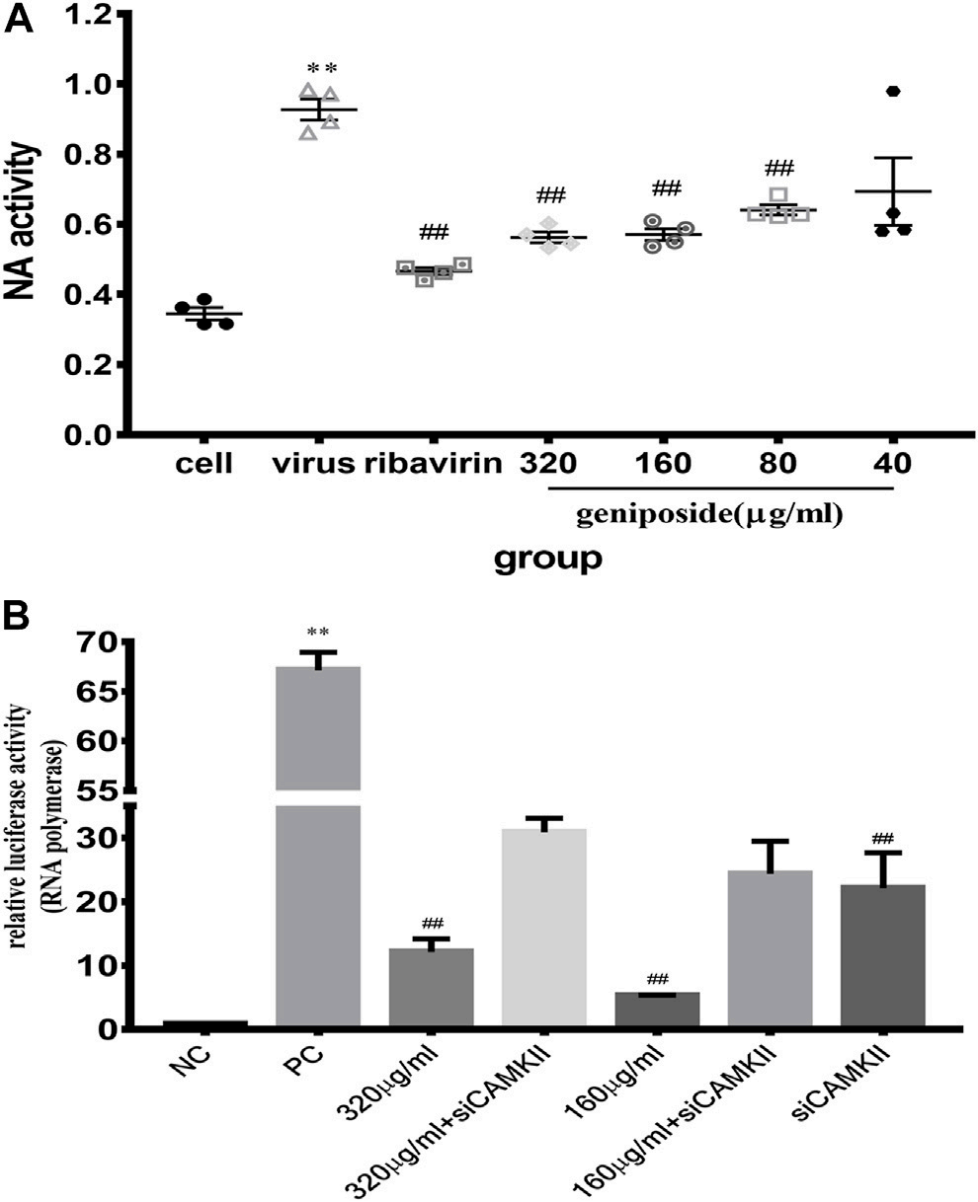
**(A)** Effect of geniposide on neuraminidase activity levels in MDCK cells infected with IAV. Values are expressed as the mean ± SEM (*n* = 4), **p < 0.01 compared to the normal control group, and ^##^ *p* < 0.01 compared to the virus control group. **(B)** Relative luciferase activity of IAV polymerase in HEK-293T cells. Relative luciferase activity was detected using a dual-luciferase reporter assay system. Values are expressed as the mean ± SEM (*n* = 4), **p < 0.01 compared to the negative control group (NC), and ^##^ *p* < 0.01 compared to the positive control group (PC).

NA plays a pivotal role during the final stages of influenza virus infection; it facilitates the release of progeny virus and the spread from infected cells to neighboring ones. To explore the inhibitory effect of geniposide on the IAV, the NA activity in MDCK cells was analyzed at 48 h post infection. In the virus control group, NA activity dramatically increased compared to the cell control group. Geniposide dramatically decreased the NA activity at concentrations of 320, 160, and 80 μg/ml, respectively (Figure 4A). The results demonstrated that geniposide significantly suppressed the IAV in MDCK cells.

### Geniposide Inhibited Polymerase Activity of IAV in HEK-293T Cells

Above, we have demonstrated the inhibitory effect of geniposide on IAV. CAMKII is one of the crucial proteins in the calcium signaling network. To clarify whether geniposide inhibited virus replication in a calcium-dependent manner, IAV polymerase activity was determined in HEK-293T cells in which CAMKII protein expression was depleted by siRNA. When CAMKII was knocked down, RNA polymerase activity was more attenuated. Therefore, the activity of RNA polymerase, which is responsible for replication and transcription, was inhibited by the knockdown of CAMKII. It was indicated that in the viral life cycle, CAMKII may promote viral proliferation. Geniposide treatment remarkably decreased IAV polymerase activity at doses of 320 and 160 μg/ml; however, IAV polymerase activity was remarkably more robust in CAMKⅡ-deficient 293T cells (Figure 4B). The results suggested that the inhibition effect of geniposide on IAV replication might be related to the calcium signaling pathway.

### Inhibitory Effect of Geniposide on the Influenza-Induced-Ca^2+^ Influx

**FIGURE 5:**
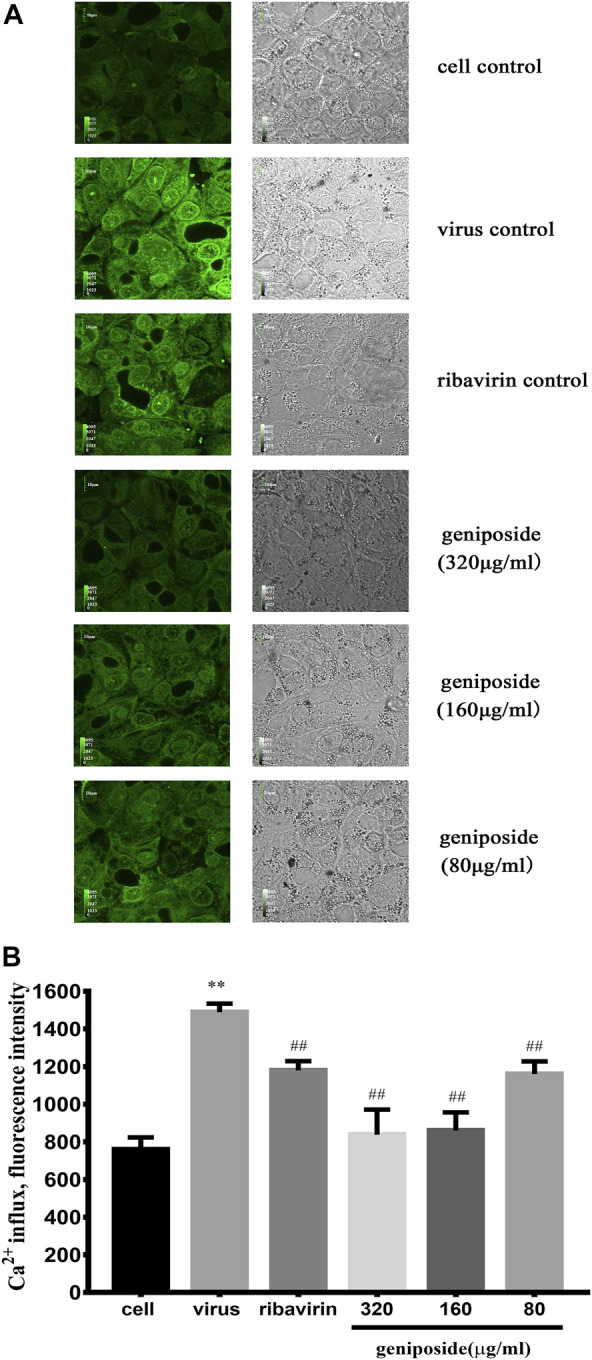
Effect of geniposide on [Ca^2+^]i levels in MDCK cells infected with influenza virus. Values were expressed as the mean fluorescence intensity of fluo-3 with four samples in each group. In each sample, 8 randomly selected fields were analyzed. **(A)** Confocal images of MDCK cells stained with fluo-3 probe at 1 h post-infection. Green images represent fluorescence images taken at emission of 530 nm. Grayscale images represent control images of MDCK cells without fluo-3 staining. **(B)** The concentration of [Ca^2+^]i was represented by mean fluorescence intensity (*n* = 4). **p < 0.01 compared to cell control group, ^##^ *p* < 0.01 compared to infection control group (A color version of this figure is available in the online journal).

Extracellular Ca^2+^ influx plays a pivotal role in IAV entry and, subsequently, mediates calcium signaling pathway activation and facilitates IAV infection in a host cell. To explore the effect of geniposide on Ca^2+^ influx in the early stage of infection, MDCK cells were stained with fluo-3 AM dye and fluorescence intensity was determined by laser scanning confocal microscopy. The results indicated that MDCK infected with IAV showed a significant increase in [Ca^2+^]_i_ at 30 min post infection. At 30 min post infection, geniposide treatment inhibited the elevation of [Ca^2+^]_i_ significantly at the concentration of 320, 160, and 80 μg/ml, which demonstrated that Ca^2+^ influx can be prevented by geniposide in the early entry step of the replication cycle of IAV (Figures 5A,B).

### Effect of Geniposide on Calcium Signaling Pathway in A549 Cells Infected by Influenza A Virus

Through network construction and functional enrichment analysis, it is shown that geniposide against influenza with the multi-target and multi-pathway mode, and calcium is one of the mechanisms. To verify the effect of geniposide on the calcium signaling pathway, the protein expression of CAMKII, CREB, and c-fos in A549 cells was determined at 24, 36, and 48 h post IAV infection.

**FIGURE 6:**
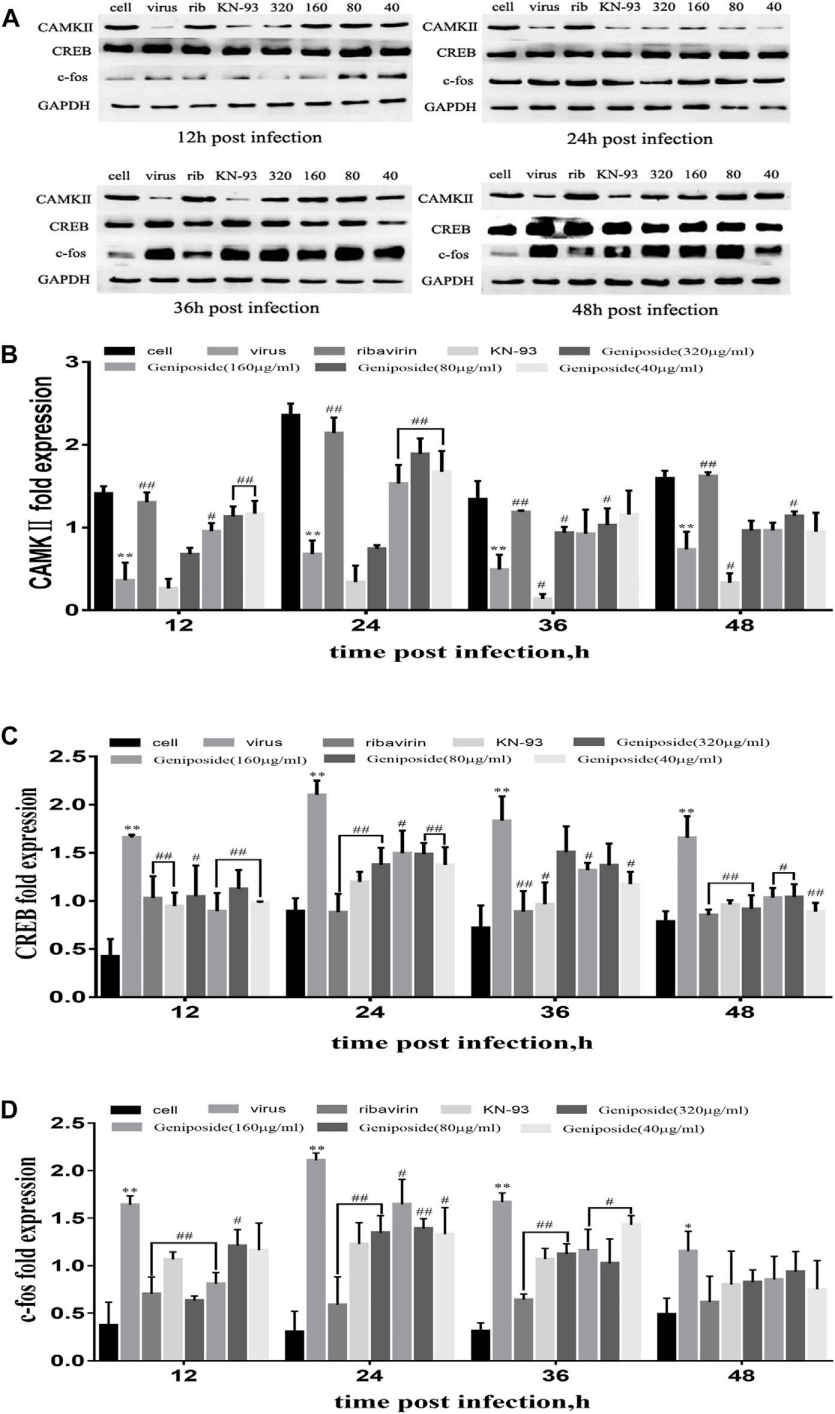
Effect of geniposide on CAMKII, CREB, and c-fos expression activated by IAV in A549 cells. Values are expressed as the mean ± SEM (*n* = 4), **p < 0.01 and *p < 0.05 compared to the normal control group, ^##^ *p* < 0.01 and ^#^ *p* < 0.05 compared to virus control group. **(A)** CAMKII, CREB, and c-fos expression were detected by Western blotting in A549 cells at 12, 24, 36, and 48 h post infection. **(B–D)** The relative fold expression of CAMKII, CREB, and c-fos was calculated by the ratio of CAMKII, CREB, and c-fos to GAPDH.

CAMKII expression was markedly decreased at 12, 24, 36, and 48 h post IAV infection. On the contrary, CREB and c-fos expressions were significantly boosted at 12, 24, 36, and 48 h post IAV infection. Geniposide treatment increased CAMKⅡ expression in a dose-dependent manner at 12, 24, 36, and 48 h post IAV infection. Furthermore, CREB and c-fos expressions were inhibited by geniposide at 12, 24, and 36 h and in all time points, respectively (Figures 6A–D). The results demonstrated that the changes in the calcium signaling pathway induced by IAV were reversed by geniposide treatment.

## Discussion

Current anti-influenza therapeutics and drugs in development are all directly targeted proteins encoded by the virus; for instance, oseltamivir inhibits NA, adamantanes block M2 ion channel, and the nucleoside analog favipiravir aims at RNA-dependent RNA polymerase (RdRP) (Goldhill et al., 2018). Alternative antiviral approaches targeting essential host proteins and pathways in the viral life cycle have great appeal because they are effective against different strains of virus and less prone to resistance. Therefore small-molecule inhibitors aiming at host factors may temporarily block the virus cycle without compromising its cellular functions.

Unique host genes have been validated as critical for influenza virus replication. For example, host genes ADAMTS7, CPE, DPP3, MST1, and PRSS12 govern inflammation (NF-κB), cAMP/calcium signaling (CRE/CREB), and apoptosis (Meliopoulos et al., 2012). Host factors BUB3, CCDC56, CLTC, CYC1, NIBP, ZC3H15, C14orf173, CTNNB1, and ANP32B showed specific inhibition effect on viral replication and transcription because they decreased the relative viral RNA polymerase activity without affecting host protein synthesis (Watanabe et al., 2014). A genome-wide CRISPR/Cas9 screen has discovered that host factors SLC35A1, C2CD4C, TRIM23, PIGN, CIC, JAK2, and PIAS3 were critical for the replication of intracellular pathogens (Han et al., 2018).

The NA of the influenza virus is a viral surface glycoprotein. As one of the most of plays pivotal role antigens of influenza virus, NA promotes the release of virions by cleaving sialic acid, and viral release and spread in the respiratory tract are facilitated (Jin et al., 2017). To explore the inhibitory effect of geniposide on IAV, the NA activity in MDCK cells was analyzed at 48 h post infection. The results demonstrated that geniposide at concentrations of different doses dramatically decreased the activity of NA that increased after virus infection, and appear in a dose-dependent manner. The results demonstrated that geniposide significantly reduced the increase of NA level after virus infection, which provides evidence that geniposide exert anti-IAV activity.

The viral RNA-dependent RNA polymerase (vRNPs) consists of vRNA, the RNA polymerase complex (RdRp), and the nuclear protein (NP), which are the smallest units of viral replication. Therefore, RNA polymerase is the key to viral replication (Li et al., 2019c). CAMKII has been identified as an antiviral host factor that interacts with IAV polymerase. The interaction between CAMKII and IAV polymerase plays a pivotal role in the outcome of virus infection and antiviral immune response. In the current study, RNA polymerase levels were remarkably decreased in the HEK-293T cells infected with IAV by geniposides that intervened. In an RNP reconstitution assay, knocking down CAMKII in IAV-infected HEK-293T cells enhanced vRNA production. The results demonstrated that the inhibition effect of geniposide on IAV replication might be related to the calcium signaling pathway after virus infection, which provides evidence that geniposide exerts anti-IAV activity.

From PPI network analysis, it is shown that 17 genes associated with geniposide hit the PPI network against the influenza virus, namely, CTLA4, AKT1, IL6, JUN, RAF1, MAPK14, CASP3, PIK3CG, FOXO1, NGF, MAPK8, BCL2, FASLG, KITLG, PTPN22, AQR, and GCG. At present, it has been reported that the host genes of geniposide that may be related to influenza virus include cytokines TNF-α, IFN-γ, IL-6, IL-4, and IL-10. Geniposide can reduce tissue damage by reducing the inflammatory cascade initiated by cytokines TNF-α and INF-γ and IL-6 (Zhang et al., 2017). Moreover, it has been reported that geniposide can enhance the protective mechanism of anti-inflammation and immune regulation by enhancing the expression of IL-4 and IL-10, so as to play an anti-influenza role (Dai et al., 2014; Chen et al., 2015a). The possible mechanism of geniposide’s inhibition on viral propagation is proved to be the downregulation of the expression of cleaved caspase 3 and reduction of caspase 3 activation. Reduce exporting ribonucleoprotein complexes (RNPs) from the nucleus, which reduces the RNPs to be packaged into infectious progeny virions at the cell membrane. This achieves the purpose of inhibiting the influenza virus (J.Wurzer et al., 2003; Chen et al., 2015b; Jiang et al., 2016).

The following genes are related to the influenza virus: CTLA4, AKT1, JUN, RAF1, PIK3CG, FOXO1, BCL2, FASLG (FASL), MAPK8, MAPK14, PTPN22, GCG, NGF, and KITLG.

According to the different functions of each gene, it can be divided into positive feedback and negative feedback to inhibit influenza virus.

Among the positive feedback genes, IAV infection affects apoptosis in early and late infection. Bcl-2 can act as an antagonist to cell death, and in late infection, it is pro-apoptotic by decreasing Bcl-2 (Mehrbod et al., 2019). Studies have shown that AKT3 induced by IAV can inhibit FoxO1, and the FoxO pathway can inhibit IAVs infection by mediating anti-apoptosis and anti-inflammatory reaction (Wu et al., 2020). MAPK8 and MAPK14 are important members of the MAPKs family, and MAP kinase controls a series of cell activities in innate immune response and participates in the regulation of cytokine gene expression and programmed cell death, which is closely related to the prevention and treatment of influenza virus (Dong et al., 2002). H1N1 infection induces an early and significant NGF upregulation. The over-expression of NGF is likely to play a neuroimmunomodulatory role in H1N1 infection. NGF acts on the nociceptive fibers innervating the lower respiratory tract, leading to enhanced neurogenic inflammation in infected lungs (Chiaretti et al., 2013). The differential genes such as KITLG, FOXP3, miR-451, IL-2, IL-10, IL-6, and TNF-α are mainly involved in viral infection and the immune-inflammatory responses. These differential genes might play a role in preventing the host from being infected by viruses and exerting immune regulatory effects in the cytoplasm (Lu et al., 2019). NA is one of the two glycoproteins on the surface of influenza virus, which cleaves terminal sialic acid residues and promotes virus release from infected cells. Studies have shown that gallic catechin gallic acid (GCG) has the best inhibitory effect on NA and affects the spread of influenza virus (Nguyen et al., 2014).

Among the negative feedback genes, the genes related to the immune system are CTLA-4, PTPN22, and FASL. CTLA-4 is a receptor on T cells, which not only plays a key role in the downregulation of the antigen-activated immune response, but also takes charge of the steady state of the immune system through regulatory T cells (Wang et al., 2001). Thus, it plays a key role in the pathogenesis of influenza virus and the defense against virus infection (Ayukawa et al., 2004). Studies have shown that PTPN22 has the function of inhibiting immune-induced T-cell expansion/activation and immune amplification of mouse T cells into peptides. PTPN22 encodes lymphoid phosphatase (Lyp), which may predispose individuals to have a diminished capacity to generate protective immunity against the influenza virus. T-cell activation contributes to the protective immunity after influenza (Hasegawa et al., 2004; Crabtree et al., 2016). Therefore, the increase of this gene is not conducive to the prevention and treatment of the influenza virus. The upregulation of Fas expression in virus-infected cells leads to the enhancement of apoptosis mediated by FasL, and Fas-FasL was involved in the apoptosis of lymphocytes induced by human influenza virus H1N1-infected monocytes through direct contact between cells (Nichols et al., 2001).

Genes with negative feedback caused by other reasons are AKT1, JUN, RAF1, and PIK3CG. IAV virus can activate Akt, trigger intracellular PI3K/Akt signaling pathway, and promote cell entry, virus protein synthesis, and virus replication (Ehrhardt et al., 2007; Shin et al., 2007). C-jun can be activated (phosphorylated) in the early stage of IAV infection (Ludwig et al., 2001); downregulation of C-jun not only significantly suppressed viral replication but also mitigated the subsequent expression of inflammatory cytokines. Therefore, C-jun plays an important role in virus infections and replications (J.Wurzer et al., 2003). By using the RNA synthesis analysis system *in vitro*, RAF-1 (RNA polymerase activator 1) and RAF-2 have been identified as host factors that can stimulate RNA synthesis of the influenza virus (Momose et al., 2001). Therefore, reducing RAF-1 can inhibit the influenza virus from the early stage.

Gene research has gradually matured, and the above genes have some functions in the anti-influenza virus. By increasing positive feedback genes and reducing negative feedback genes, the influenza virus can be prevented and treated.

However, there is no evidence that AQR is related to the influenza virus after a keyword search.

From module enrichment analysis, the primary GO biological processes and KEGG pathways were identified. The primary GO biological processes identified were involved in cell communication (14.53%), metabolism (13.41%), transport (11.17%), signal transduction (9.5%), response external stimulus (5.03%), and viral life cycle (0.84%), which might be the main mechanisms of geniposide against influenza virus. Furthermore, nine GO biological processes were related to the function of calcium ion and signaling pathway, namely, cellular calcium ion homeostasis, calcium ion homeostasis, cytosolic calcium ion homeostasis, the elevation of cytosolic calcium ion concentration, regulation of calcium ion transport, elevation of cytosolic calcium ion concentration during G-protein signaling, negative regulation of calcium ion transport *via* voltage-gated calcium channel activity, regulation of calcium ion transport *via* voltage-gated calcium channel activity, and negative regulation of calcium ion transport 3, which belong to cell homeostasis, cell communication, transport, ion transport, and signal transduction. The results indicated that calcium ions might play a pivotal role in geniposide inhibiting the influenza virus.

Calcium/calmodulin-dependent protein kinase (CaM kinase) II (CAMKII) is one of the crucial proteins in the calcium signaling network. Calcium signaling which is activated by Ca^2+^ is a key regulator of IAV internalization and infection, IAV is shown to cause Ca^2+^ influx. Calcium signaling is a key regulator of IAV internalization and is a ubiquitously expressed calcium sensor that regulates diverse cellular functions. CAMK II was confirmed to be involved in post-entry steps of influenza virus replication by genome-wide RNAi screens. A recent study highlighted that diltiazem, a calcium channel blocker, significantly inhibited viral production in human lung epithelial A549 cells (*in vitro*) and (*ex vivo*) reconstituted the human airway epithelium (HAE) model. Moreover, diltiazem treatment remarkably prevented mortality and reduced weight loss in mice infected with influenza A (H1N1) pdm09, as a promising candidate for the treatment of influenza infections (Pizzorno et al., 2019). Repurposing of drugs as novel influenza inhibitors from clinical gene expression infection signatures.

From functional enrichment analysis, calcium signaling pathway was identified in module 1, and geniposide may play an anti-influenza virus role by inhibiting calcium influx.

To clarify whether geniposide inhibited virus replication in a calcium-dependent manner, the activity of RNA polymerase, which is responsible for replication and transcription, was inhibited by knockdown of CAMKII. It was indicated that in the viral life cycle, CAMKII may promote viral proliferation. Geniposide treatment remarkably decreased IAV polymerase activity. However, IAV polymerase activity was remarkably more robust in CAMKII-deficient 293T cells. The results suggested that the inhibition effect of geniposide on IAV replication might be related to the calcium signaling pathway.

Extracellular Ca^2+^ influx plays a key role in the invasion of IAV, which, in turn, mediates the activation of the calcium signaling pathway and promotes host cell IAV infection. To further investigate whether geniposide suppresses IAV replication by decreasing extracellular Ca^2+^ influx, MDCK cells were stained with fluo-3 AM dye and fluorescence intensity was determined by laser scanning confocal microscopy. According to the results of the fluorescence intensity assay, geniposide significantly inhibited the increase of [Ca^2+^]i in MDCK infected with IAV 30 min after infection, indicating that geniposide prevented Ca^2+^ influx at the early stage of the IAV replication cycle to exert an antiviral effect.

CAMK cascade is important for many normal physiological processes that when misregulated can lead to a variety of disease states of cell proliferation and apoptosis (Colomer and Means, 2007). Numerous studies have linked the transcription factor CREB to modulation of various inflammatory mediators. CREB signaling is an important cellular process that serves a variety of functions. Most notably with respect to influenza infection, CREB signaling has been shown to activate protein kinase A (PKA) and thus have a role in protein synthesis (Meliopoulos et al., 2012; Andrews et al., 2015). c-fos, as a CREB downstream expression factor, also plays an important role in the virus infection. Studies have shown that c-fos expression was significantly increased after virus infections. Upon investigation of the underlying mechanisms, c-fos transcriptional activating protein was demonstrated to activate the IL-6 and IL-8 promoters (Moreno et al., 2011; Peng et al., 2016). CAMKII induced the expression of downstream factor CREB, and c-fos plays a crucial role in Ca^2+^ influx post-IAV infection. CaMKII activation leads to the inhibition of the downstream factor CREB and c-fos activity, which results in the arrest of virus replication. It was catalyzed by repressed IAV polymerase, which is an efficient way to suppress virus replication. Geniposide on Ca^2+^ signal transduction pathway may be related to the inhibition of the CaMKII downstream factor CREB and c-fos activity.

To verify the inhibition of CaMKII and the activation of CREB and c-fos induced by IAV infection, we measured CAMKII, CREB, and c-fos expression in infected A549 cells 12, 24, 36, and 48 h post-infection. Geniposide treatment markedly increased CAMKII expression at the four measurement time points post-infection. Furthermore, CREB and c-fos expressions were inhibited by geniposide in all time points, respectively. These results indicated that the suppression of CAMKII and over-activation of CREB and c-fos by IAV were changed by geniposide treatment, which provides evidence that geniposide exerts anti-IAV activity *via* the changes of calcium signaling pathway induced by IAV, and relevant results of module enrichment analysis were verified.

Collectively, the data from the current study were consistent with the PPI network analysis results. In conclusion, our research indicated that geniposide sufficiently suppressed IAV replication *in vitro*. These anti-IAV effects may be directly related to the inhibition of viral proliferation by host factors, which inhibited virus replication in a CAMKII-dependent replication manner, preventing the over-activation of IAV polymerase response induced by IAV infection. Taken together, our findings reveal a new facet of the mechanism of geniposide action against the IAV, for which it inhibited IAV replication in disrupted interplay between IAV RNA polymerase and CAMKII and the regulation on changes of calcium signaling pathway essential for IAV replication. The current study might pave the way for the development of new antiviral agents against the influenza virus.

## Data Availability

The original contributions presented in the study are included in the article/Supplementary Material. Further inquiries can be directed to the corresponding authors.
