# Supplementary figures and images for: An Integrated Analysis Reveals Geniposide Extracted From Gardenia jasminoides J.Ellis Regulates Calcium Signaling Pathway Essential for Influenza A Virus Replication
Source: Front Pharmacol. 2021 Nov 19;12:755796. doi: 10.3389/fphar.2021.755796 (PMC8640456; doi:10.3389/fphar.2021.755796)

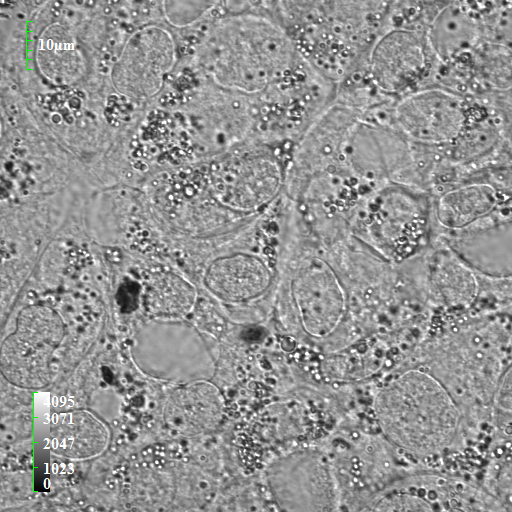

Supplement: Supplementary file 1 [file DataSheet3.ZIP › fig5/geniposide80.tif.frames/geniposide80_C002.tif]

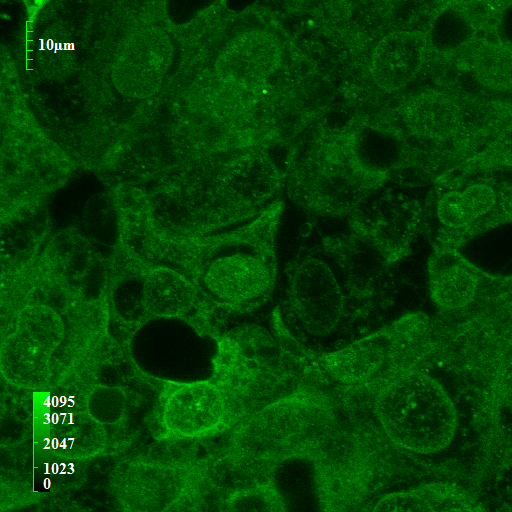

Supplement: Supplementary file 1 [file DataSheet3.ZIP › fig5/geniposide80.tif.frames/geniposide80_C001.tif]

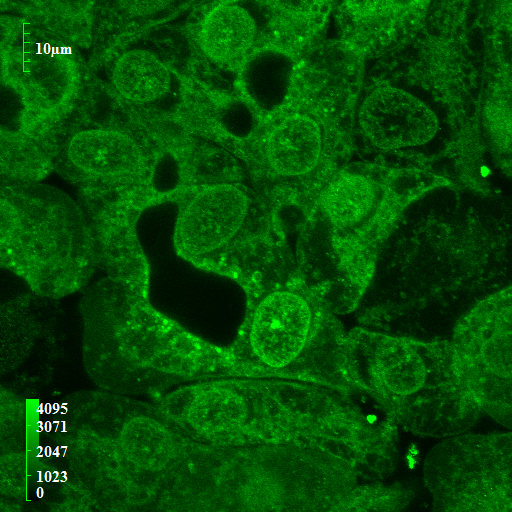

Supplement: Supplementary file 1 [file DataSheet3.ZIP › fig5/ribavirin.tif.frames/ribavirin_C001.tif]

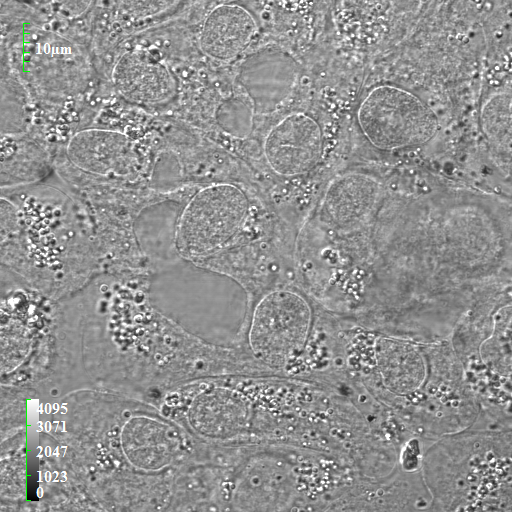

Supplement: Supplementary file 1 [file DataSheet3.ZIP › fig5/ribavirin.tif.frames/ribavirin_C002.tif]

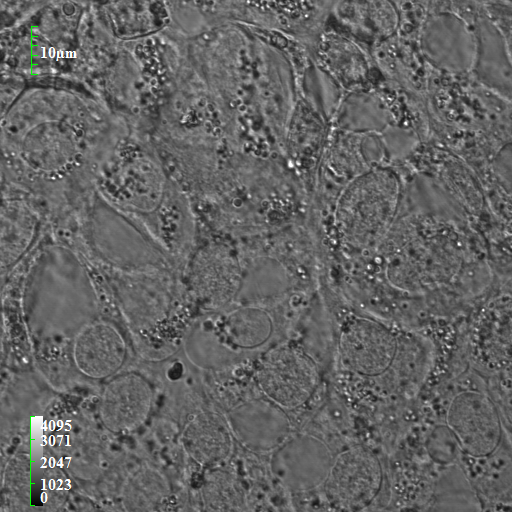

Supplement: Supplementary file 1 [file DataSheet3.ZIP › fig5/geniposide320.tif.frames/geniposide160_C002.tif]

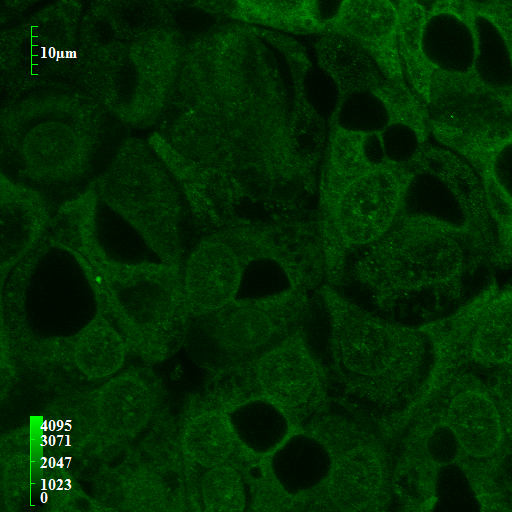

Supplement: Supplementary file 1 [file DataSheet3.ZIP › fig5/geniposide320.tif.frames/geniposide160_C001.tif]

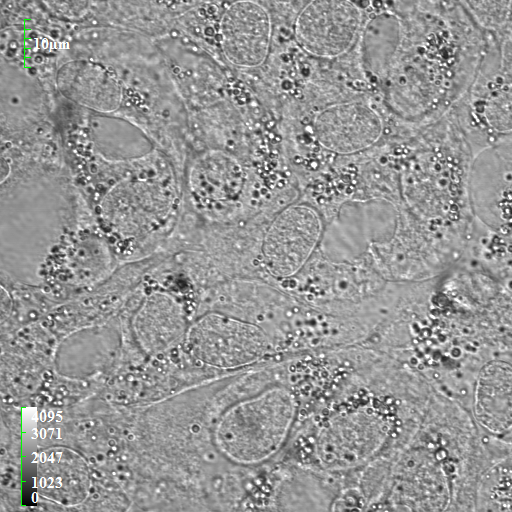

Supplement: Supplementary file 1 [file DataSheet3.ZIP › fig5/Geniposide160.tif.frames/Geniposide320_C002.tif]

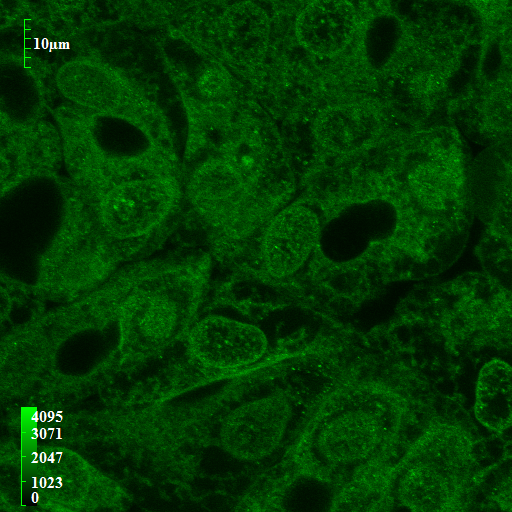

Supplement: Supplementary file 1 [file DataSheet3.ZIP › fig5/Geniposide160.tif.frames/Geniposide320_C001.tif]

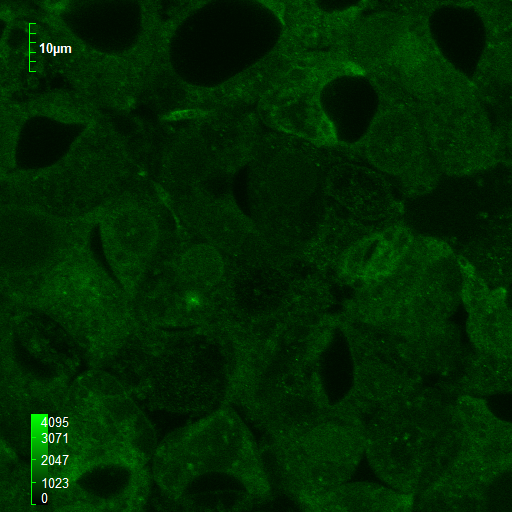

Supplement: Supplementary file 1 [file DataSheet3.ZIP › fig5/N.tif.frames/N_C001.tif]

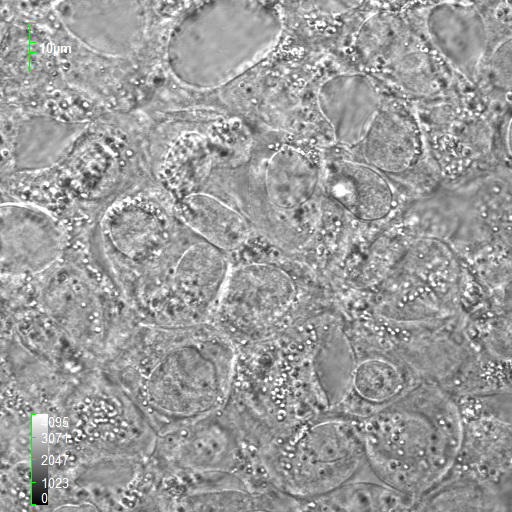

Supplement: Supplementary file 1 [file DataSheet3.ZIP › fig5/N.tif.frames/N_C002.tif]

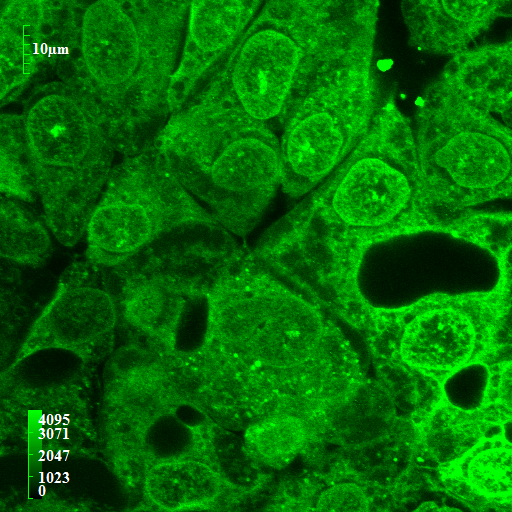

Supplement: Supplementary file 1 [file DataSheet3.ZIP › fig5/FM1.tif.frames/FM1_C001.tif]

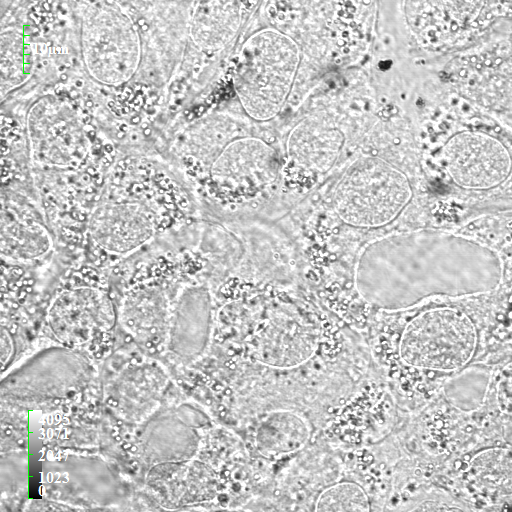

Supplement: Supplementary file 1 [file DataSheet3.ZIP › fig5/FM1.tif.frames/FM1_C002.tif]

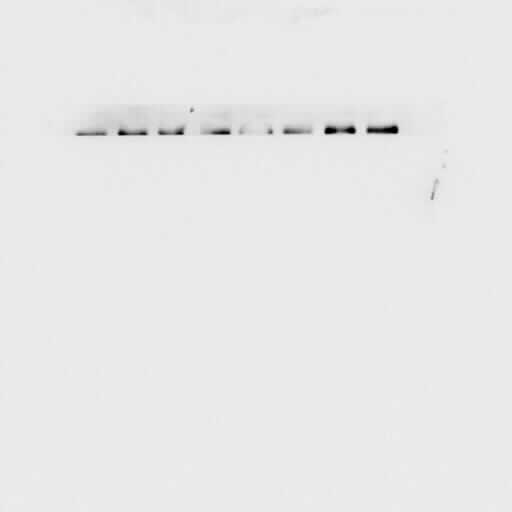

Supplement: Supplementary file 5 [file DataSheet1.ZIP › camk/12 c-fos.JPG]

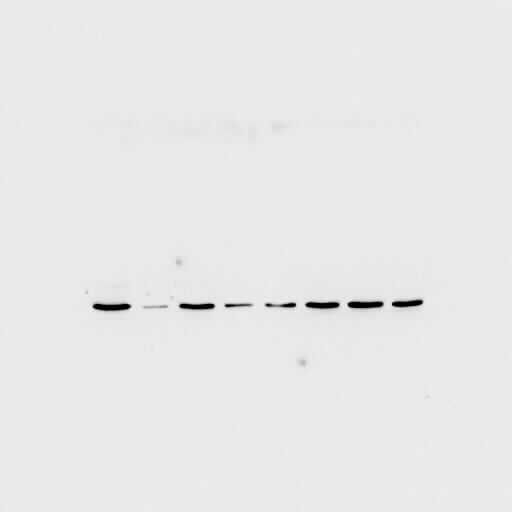

Supplement: Supplementary file 5 [file DataSheet1.ZIP › camk/12 camk.JPG]

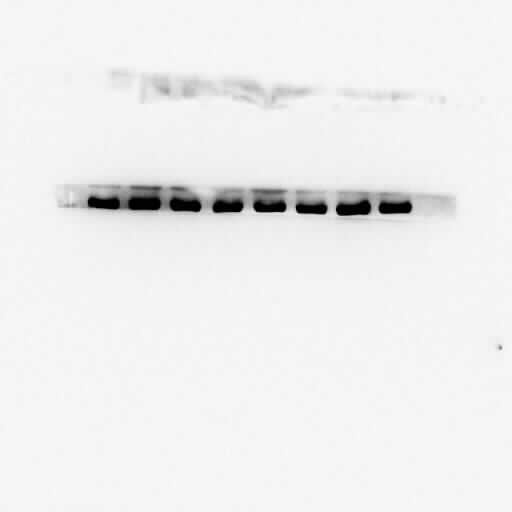

Supplement: Supplementary file 5 [file DataSheet1.ZIP › camk/12 creb.JPG]

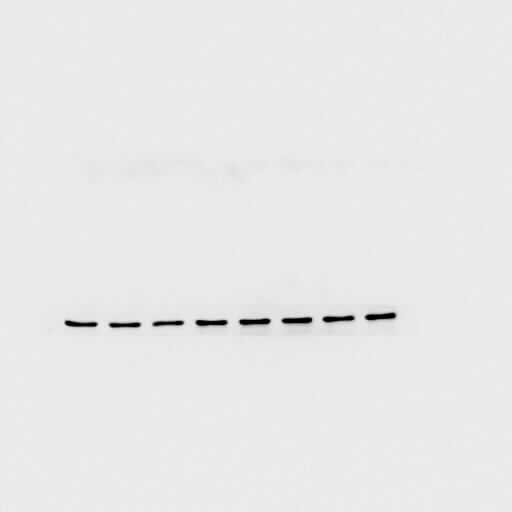

Supplement: Supplementary file 5 [file DataSheet1.ZIP › camk/12 gapdh.JPG]

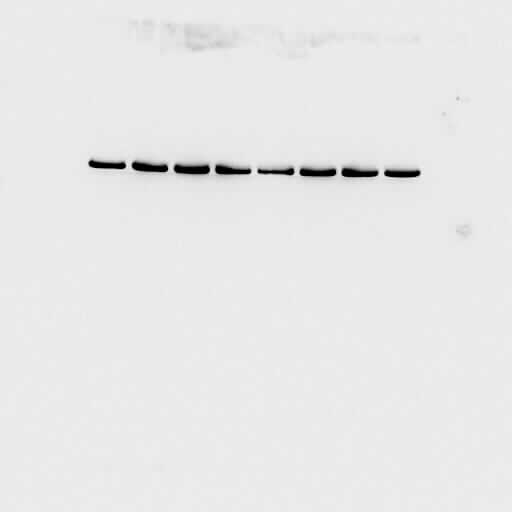

Supplement: Supplementary file 5 [file DataSheet1.ZIP › camk/24 c-fos.JPG]

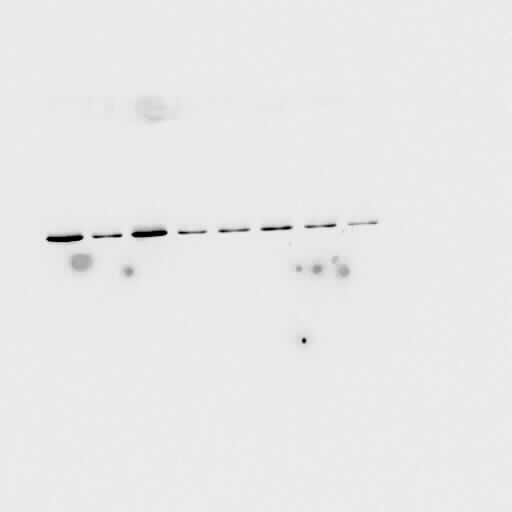

Supplement: Supplementary file 5 [file DataSheet1.ZIP › camk/24 camk.JPG]

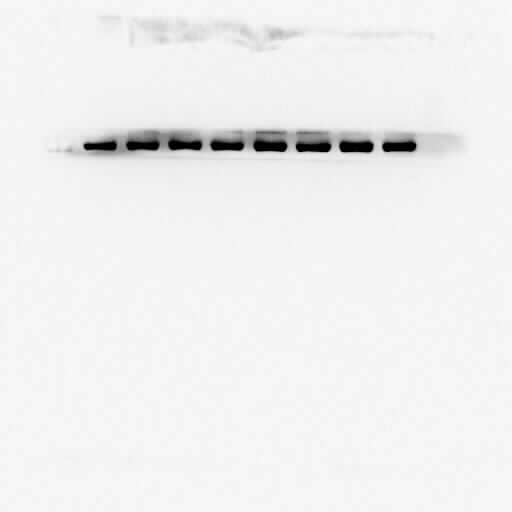

Supplement: Supplementary file 5 [file DataSheet1.ZIP › camk/24 creb.JPG]

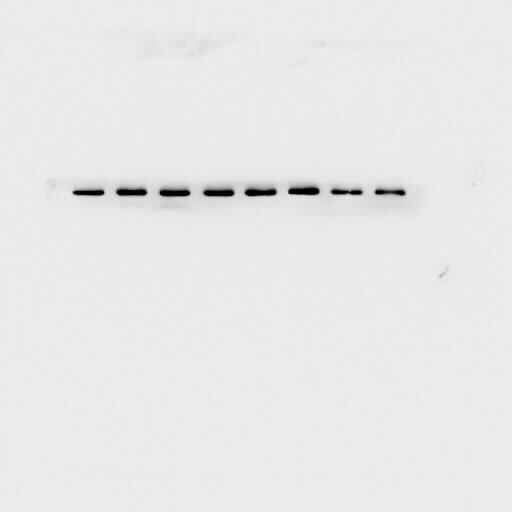

Supplement: Supplementary file 5 [file DataSheet1.ZIP › camk/24 gapdh.JPG]

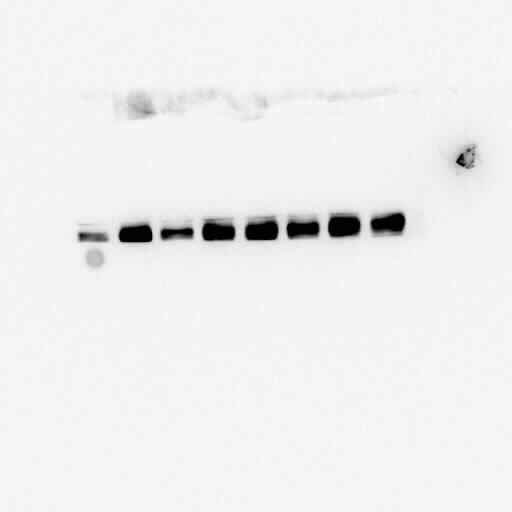

Supplement: Supplementary file 5 [file DataSheet1.ZIP › camk/36 c-fos.JPG]

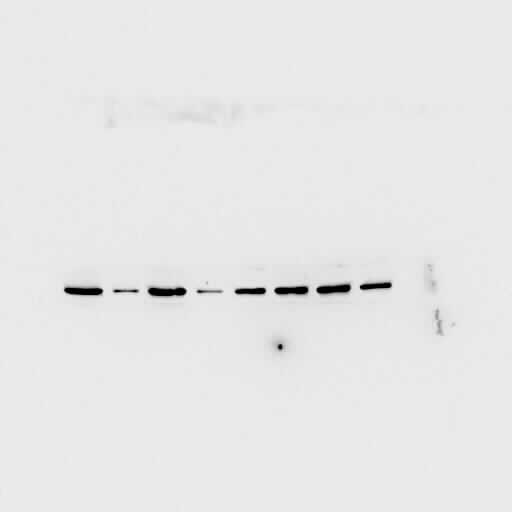

Supplement: Supplementary file 5 [file DataSheet1.ZIP › camk/36 camk.JPG]

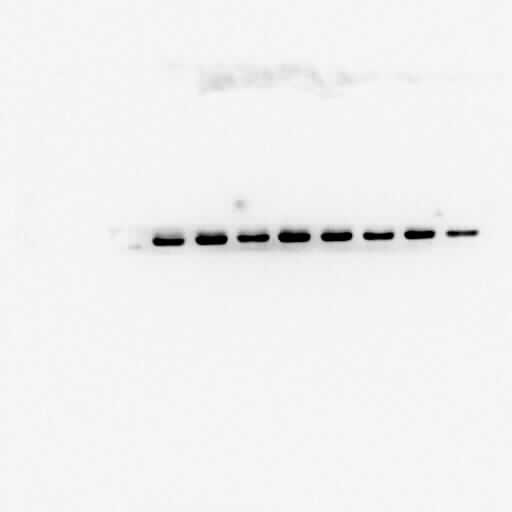

Supplement: Supplementary file 5 [file DataSheet1.ZIP › camk/36 creb.JPG]

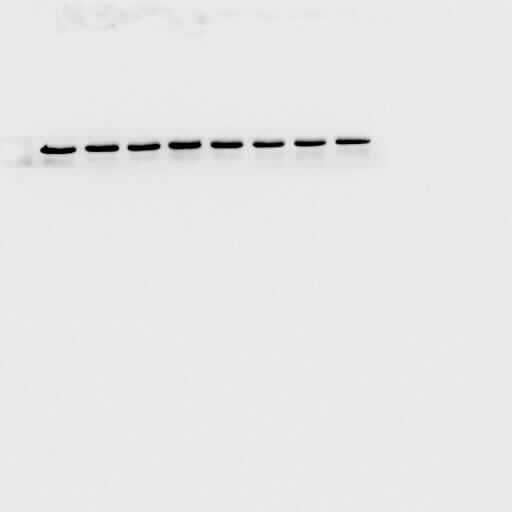

Supplement: Supplementary file 5 [file DataSheet1.ZIP › camk/36 gapdh.JPG]

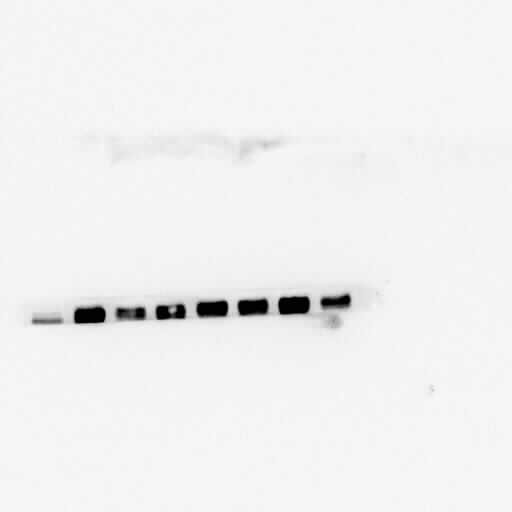

Supplement: Supplementary file 5 [file DataSheet1.ZIP › camk/48 c-fos.JPG]

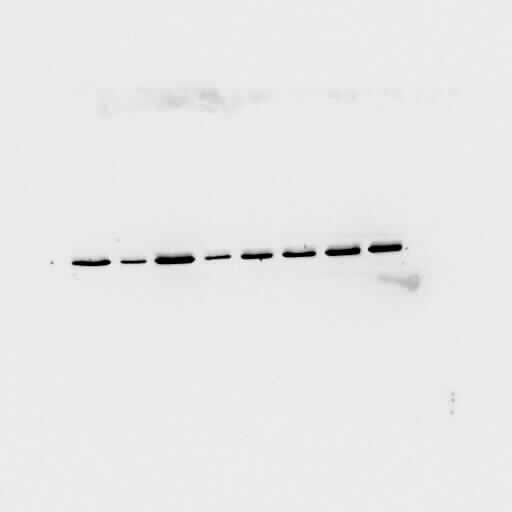

Supplement: Supplementary file 5 [file DataSheet1.ZIP › camk/48 camk.JPG]

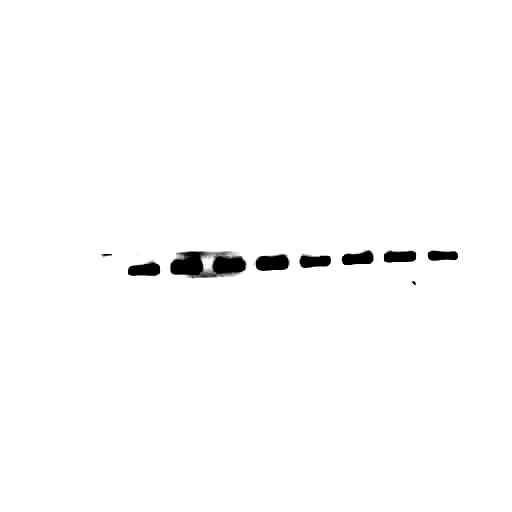

Supplement: Supplementary file 5 [file DataSheet1.ZIP › camk/48 creb.jpg]

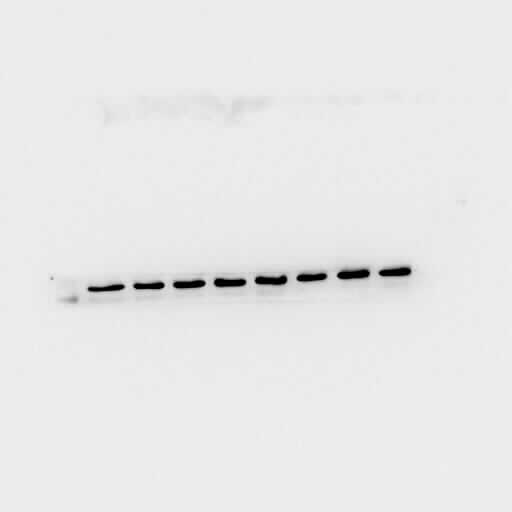

Supplement: Supplementary file 5 [file DataSheet1.ZIP › camk/48 gapdh.JPG]
