# Supplementary material for: An Integrated Analysis Reveals Geniposide Extracted From Gardenia jasminoides J.Ellis Regulates Calcium Signaling Pathway Essential for Influenza A Virus Replication
Source: Front Pharmacol. 2021 Nov 19;12:755796. doi: 10.3389/fphar.2021.755796 (PMC8640456; doi:10.3389/fphar.2021.755796)
Supplement: Supplementary file 4 [file DataSheet4.ZIP › μáÇσ¡ÉΦï╖/5ul.pdf]

色谱图和结果

进样信息

进样名称:	**5UL栀子苷**	*保留时间 (min):*	**20.00**
瓶号:	**RD2**	*进样量:*	**5.00**
进样类型:	**Unknown**	*通道:*	**UV_VIS_1**
校准级别:		*波长:*	**238.0**
仪器方法:	**15%乙腈+85%水等度20min**	*带宽:*	**4**
处理方法:	处理方法	*稀释因子:*	**1.0000**
进样日期/时间:	**19/一月/16 17:54**	*样品重量:*	**1.0000**

色谱图

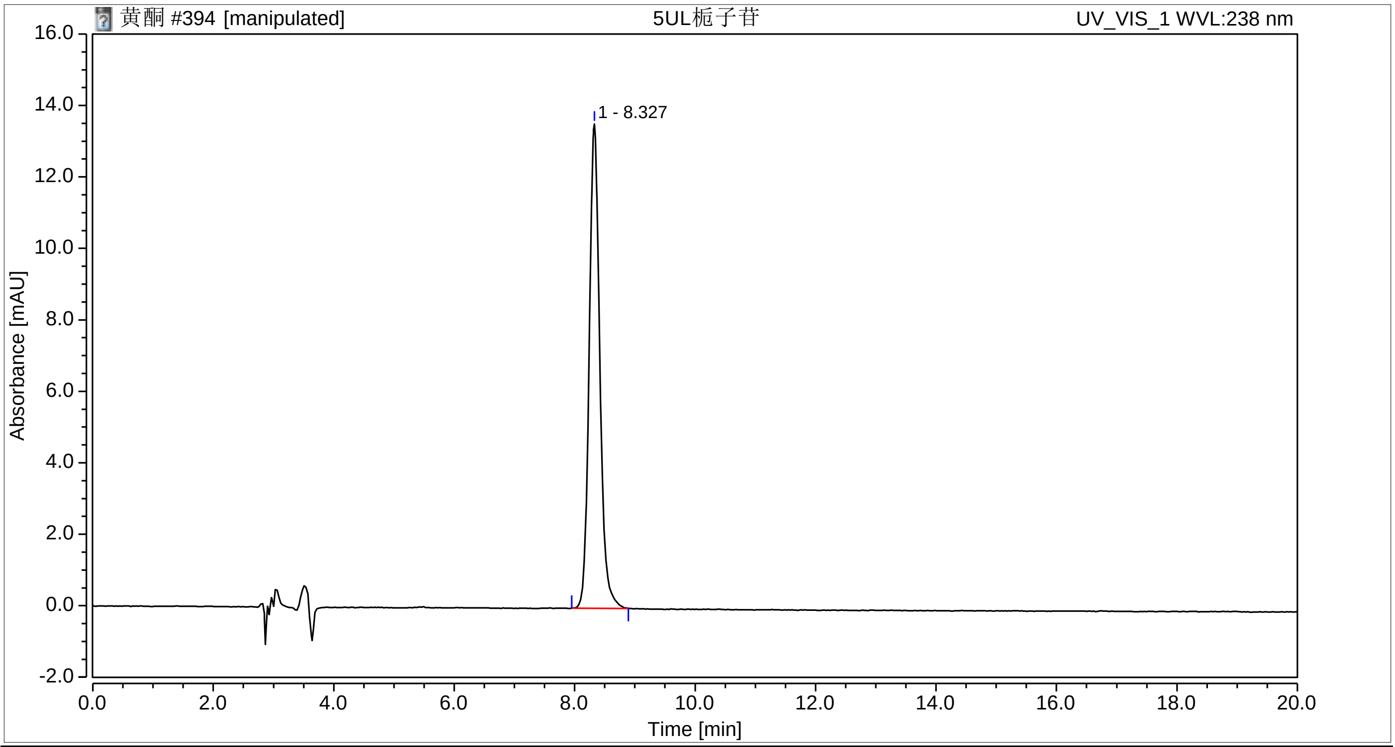

积分结果

序号	峰名称	保留时间<br>min	峰面积<br>mAU*min	峰高<br>mAU	相对峰面积<br>%	相对峰高<br>%	样品量<br>n.a.
1		8.327	2.767	13.559	100.00	100.00	n.a.
总和:			**2.767**	**13.559**	**100.00**	**100.00**	

色谱图和 SST 结果

进样信息

进样名称:	**5UL栀子苷**	运行时间 (min):	**20.00**
瓶号:	**RD2**	进样量:	**5.00**
进样类型:	**Unknown**	通道:	**UV_VIS_1**
校准级别:		波长:	**238.0**
仪器方法:	**15%乙腈+85%水等度20min**	带宽:	**4**
处理方法:	**处理方法**	稀释因子:	**1.0000**
进样日期/时间:	**19/一月/16 17:54**	样品重量:	**1.0000**

色谱图

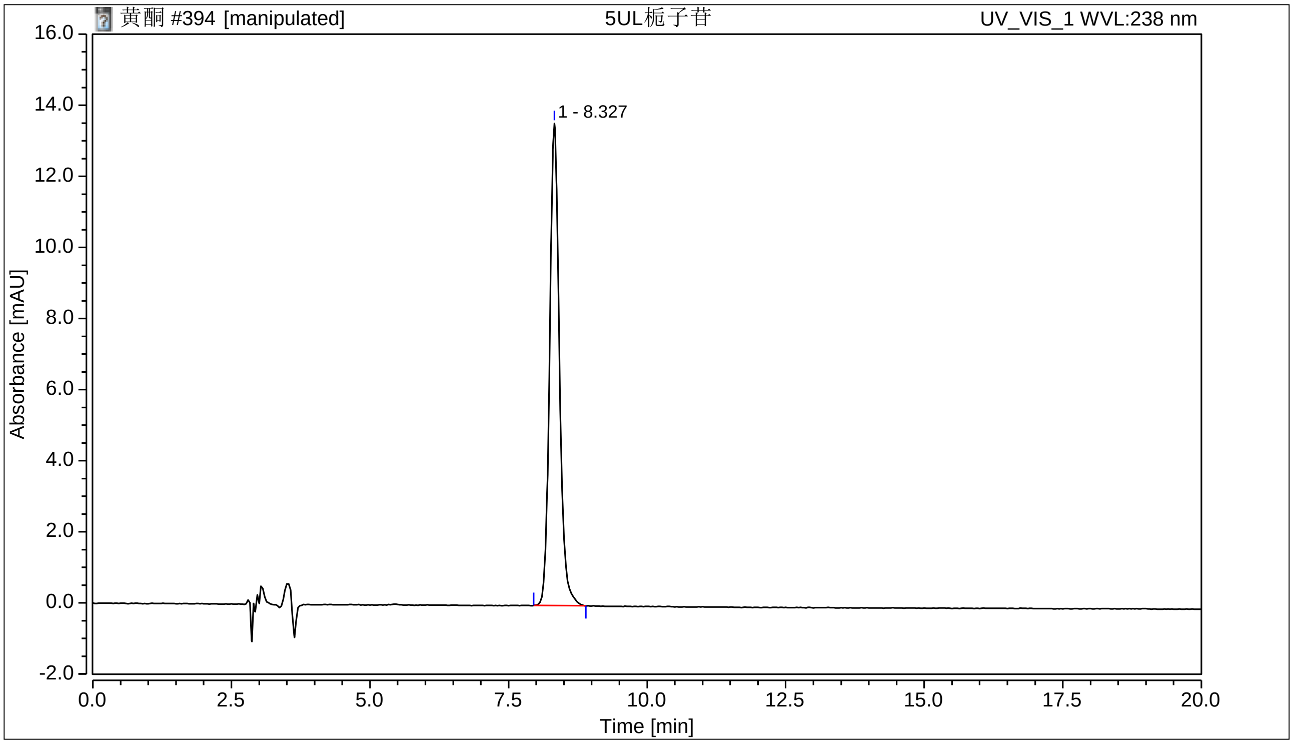

SST 结果

序号	名称	进样条件	色谱峰	测试结果	进样
	已执行的测试用例数量:	**n.a.**	总结果:	通过	

光谱结果

进样信息

进样名称:	**5UL栀子苷**	*运行时间 (min):*	**20.00**
瓶号:	**RD2**	*进样量:*	**5.00**
进样类型:	**Unknown**	*通道:*	**UV_VIS_1**
校准级别:		*波长:*	**238.0**
仪器方法:	**15%乙腈+85%水等度20min**	*带宽:*	**4**
处理方法:	**处理方法**	*稀释因子:*	**1.0000**
进样日期/时间:	**19/一月/16 17:54**	*样品重量:*	**1.0000**

等值图

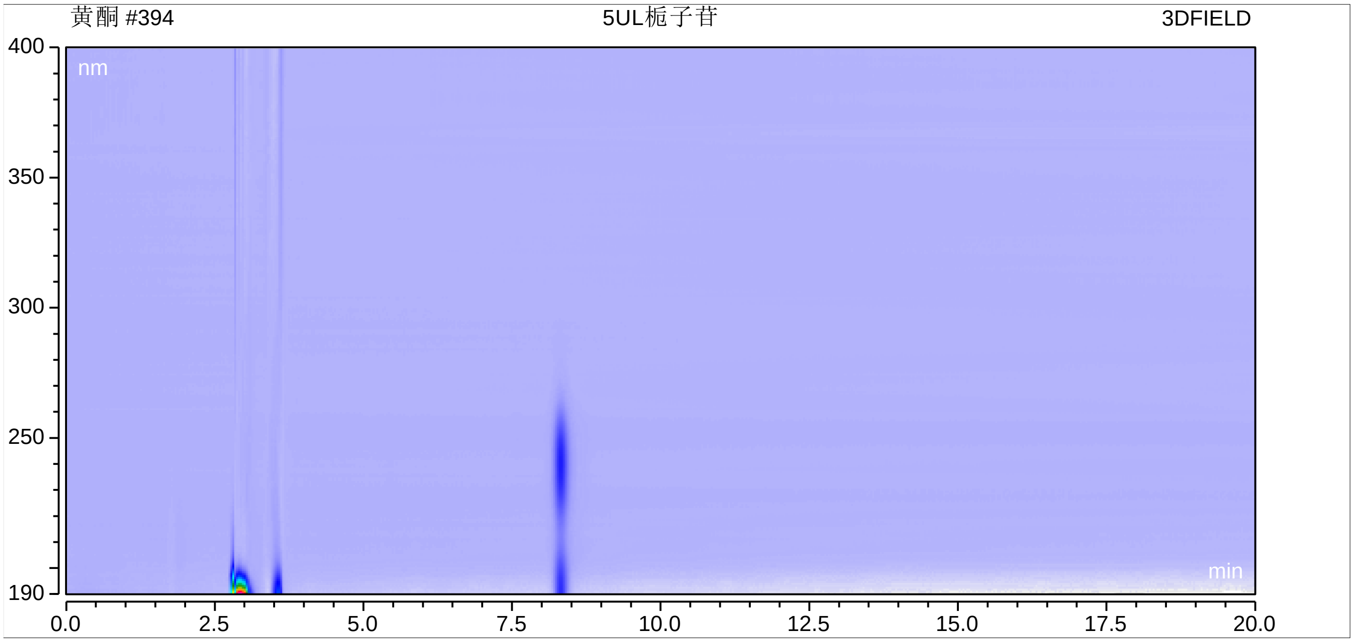

峰纯度

序号	峰名称	保留时间<br>min	匹配	RSD 匹配<br>%	PPI<br>nm	RSD PPI<br>%	n极值

光谱库筛选结果

进样信息

进样名称:	**5UL栀子苷**	*运行时间 (min):*	**20.00**
瓶号:	**RD2**	*进样量:*	**5.00**
进样类型:	**Unknown**	*通道:*	**UV_VIS_1**
校准级别:		*波长:*	**238.0**
仪器方法:	**15%乙腈+85%水等度20min**	*带宽:*	**4**
处理方法:	处理方法	*稀释因子:*	**1.0000**
进样日期/时间:	**19/一月/16 17:54**	*样品重量:*	**1.0000**

UV 光谱 n.a.

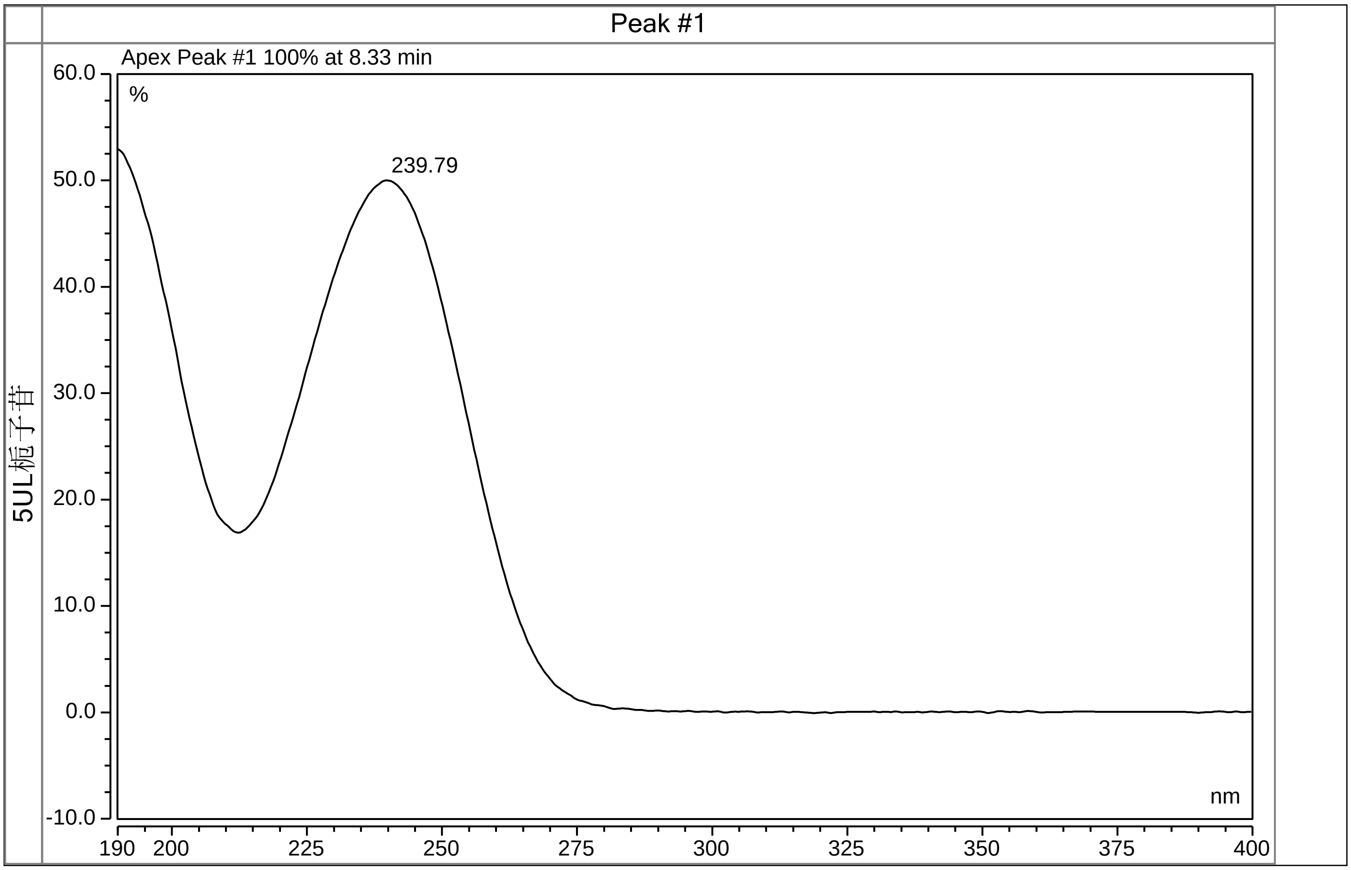

光谱库筛选结果

	匹配# 1	匹配# 2	匹配# 3
库:	n.a.	n.a.	n.a.
组份:	n.a.	n.a.	n.a.
匹配因子:	n.a.	n.a.	n.a.
保留时间:	n.a.	n.a.	n.a.
来源:			
采集日期:	n.a.	n.a.	n.a.
仪器方法:	n.a.	n.a.	n.a.
