# Supplementary material for: An Integrated Analysis Reveals Geniposide Extracted From Gardenia jasminoides J.Ellis Regulates Calcium Signaling Pathway Essential for Influenza A Virus Replication
Source: Front Pharmacol. 2021 Nov 19;12:755796. doi: 10.3389/fphar.2021.755796 (PMC8640456; doi:10.3389/fphar.2021.755796)
Supplement: Supplementary file 4 [file DataSheet4.ZIP › μáÇσ¡ÉΦï╖/gss.pdf]

色谱图和结果

进样信息

进样名称:	**1.22mg/25mLGSS**	*保留时间 (min):*	**40.00**
瓶号:	**RD1**	*进样量:*	**10.00**
进样类型:	**Unknown**	*通道:*	**UV_VIS_1**
校准级别:		*波长:*	**238.0**
仪器方法:	**15%乙腈+85%水等度洗脱40min**	*带宽:*	**4**
处理方法:	处理方法	*稀释因子:*	**1.0000**
进样日期/时间:	**19/一月/16 14:08**	*样品重量:*	**1.0000**

色谱图

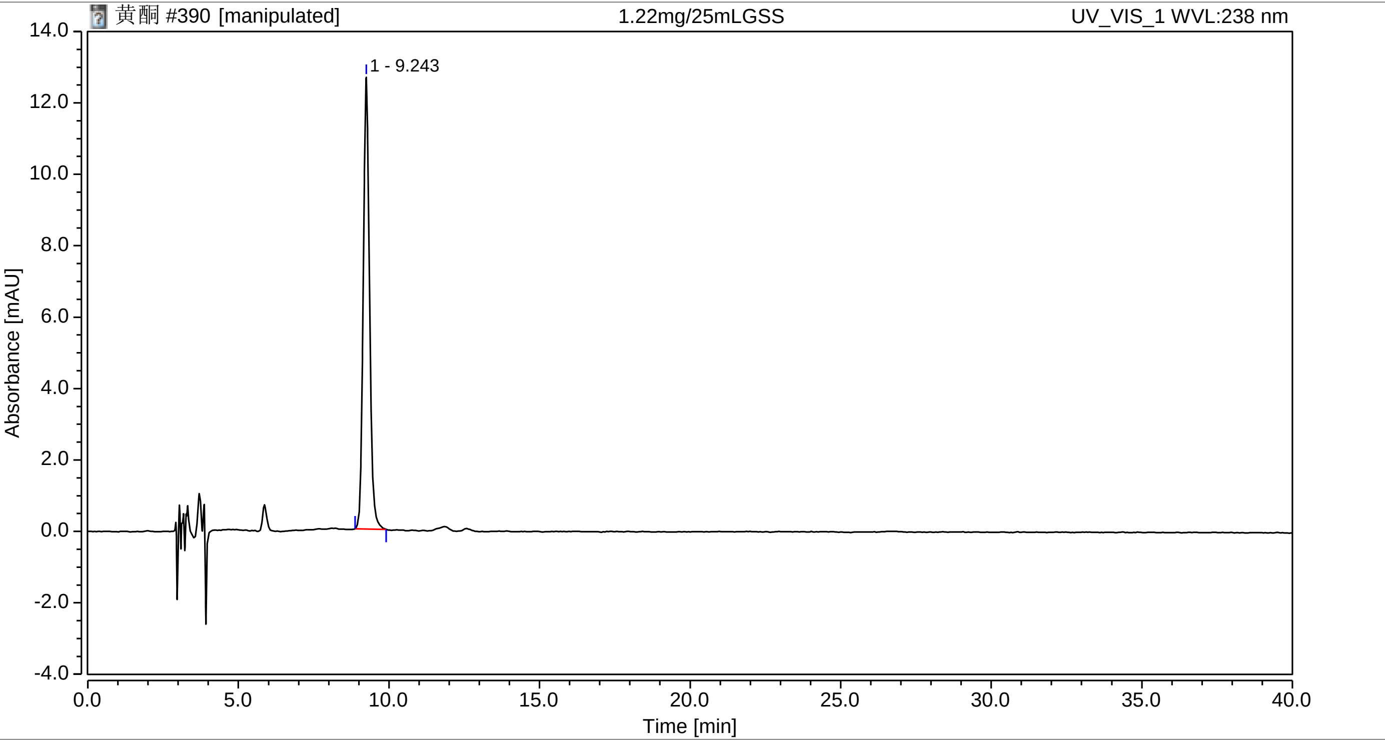

积分结果

序号	峰名称	保留时间<br>min	峰面积<br>mAU*min	峰高<br>mAU	相对峰面积<br>%	相对峰高<br>%	样品量<br>n.a.
1		9.243	3.046	12.651	100.00	100.00	n.a.
总和:			**3.046**	**12.651**	**100.00**	**100.00**	

Chromeleon (c) Dionex
版本 7.2.0.3765

色谱图和 SST 结果

进样信息

进样名称:	**1.22mg/25mLGSS**	*运行时间 (min):*	**40.00**
瓶号:	**RD1**	*进样量:*	**10.00**
进样类型:	**Unknown**	*通道:*	**UV_VIS_1**
校准级别:		*波长:*	**238.0**
仪器方法:	**15%乙腈+85%水等度洗脱40min**	*带宽:*	**4**
处理方法:	处理方法	*稀释因子:*	**1.0000**
进样日期/时间:	**19/一月/16 14:08**	*样品重量:*	**1.0000**

色谱图

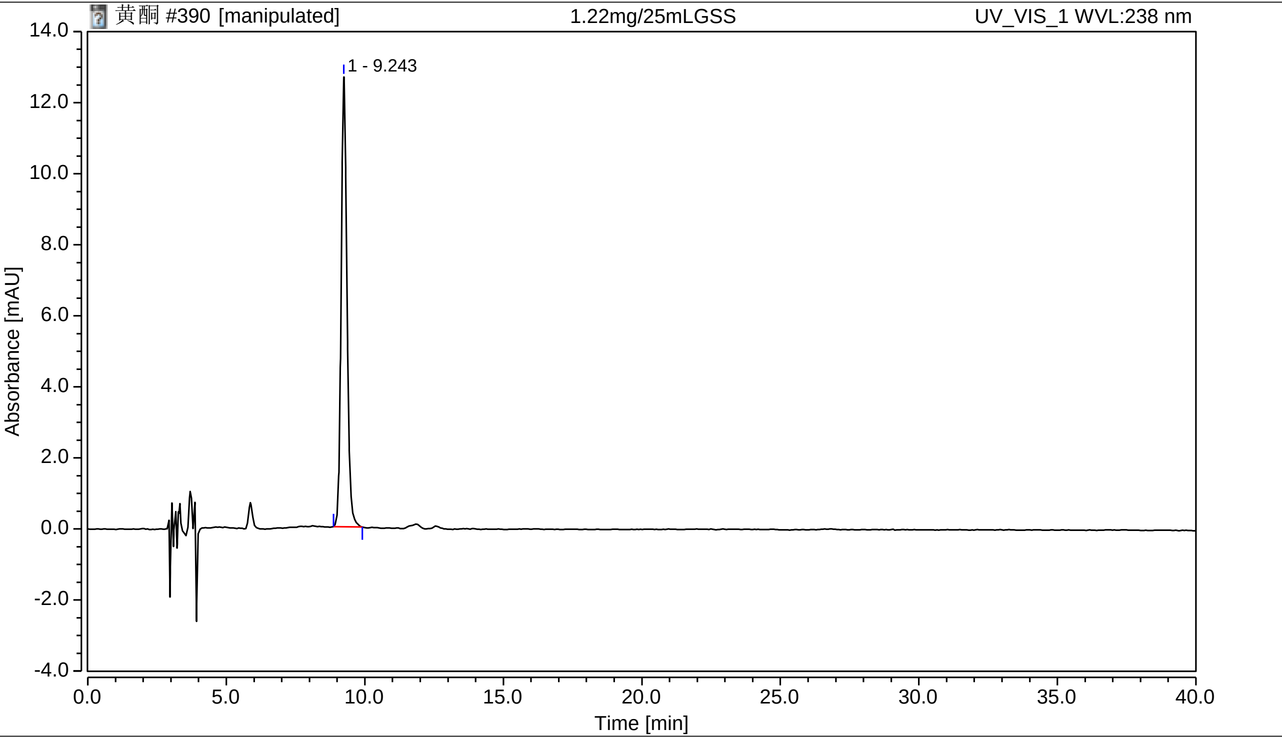

SST 结果

序号	名称	进样条件	色谱峰	测试结果	进样
	已执行的测试用例数量:	n.a.	总结果:	通过	

光谱结果

进样信息

进样名称:	**1.22mg/25mLGSS**	*运行时间 (min):*	**40.00**
瓶号:	**RD1**	*进样量:*	**10.00**
进样类型:	**Unknown**	*通道:*	**UV_VIS_1**
校准级别:		*波长:*	**238.0**
仪器方法:	**15%乙腈+85%水等度洗脱40min**	*带宽:*	**4**
处理方法:	处理方法	*稀释因子:*	**1.0000**
进样日期/时间:	**19/一月/16 14:08**	*样品重量:*	**1.0000**

等值图

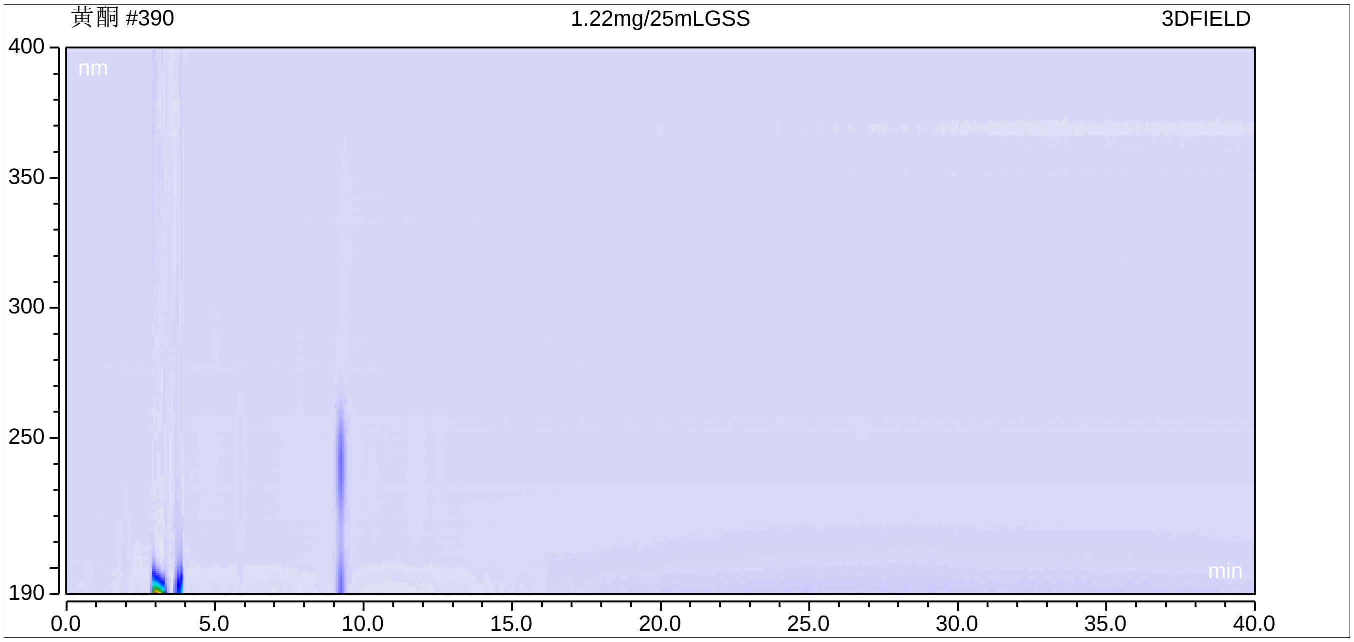

峰纯度

序号	峰名称	保留时间 min	匹配	RSD 匹配 %	PPI nm	RSD PPI %	n极值

光谱库筛选结果

进样信息

进样名称:	1.22mg/25mLGSS	运行时间 (min):	40.00
瓶号:	RD1	进样量:	10.00
进样类型:	Unknown	通道:	UV_VIS_1
校准级别:		波长:	238.0
仪器方法:	15%乙腈+85%水等度洗脱40min	带宽:	4
处理方法:	处理方法	稀释因子:	1.0000
进样日期/时间:	19/一月/16 14:08	样品重量:	1.0000

UV 光谱 n.a.

Peak #1

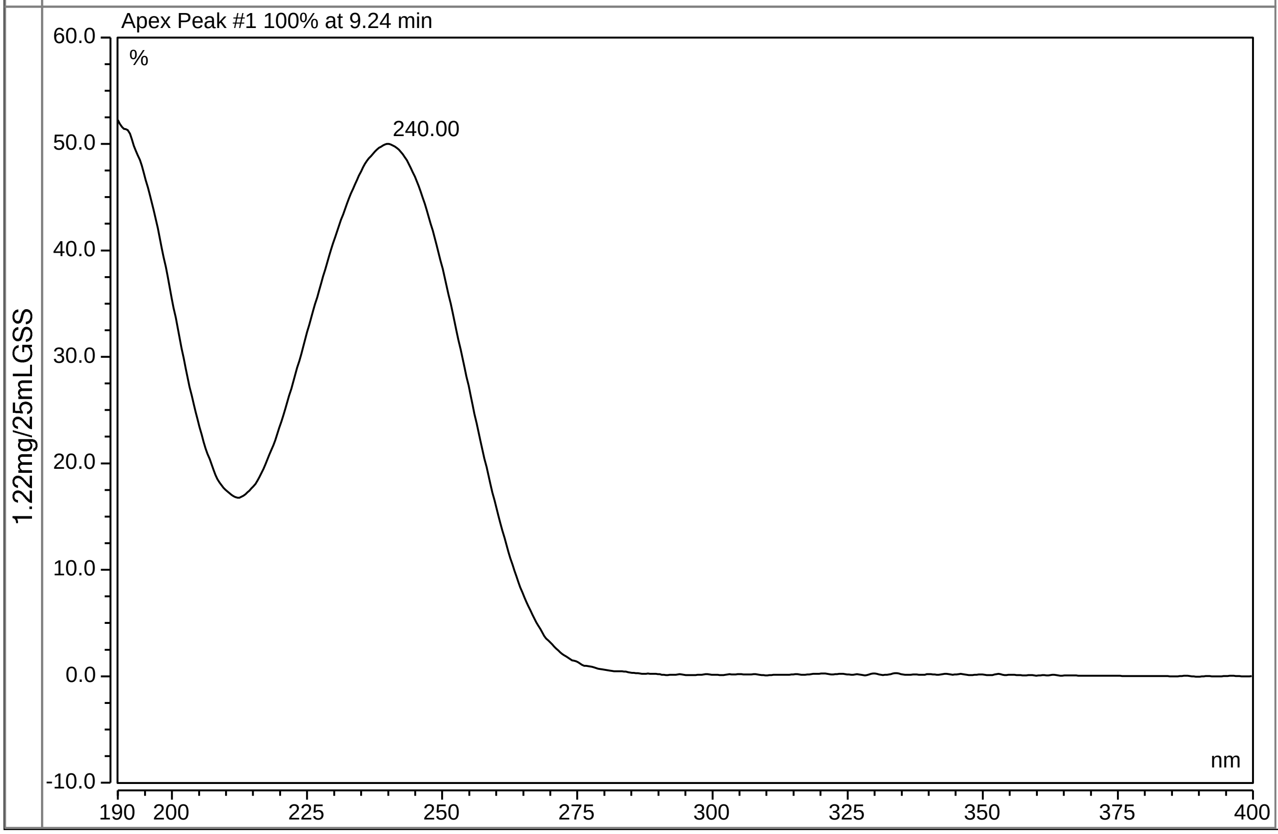

光谱库筛选结果

	匹配# 1	匹配# 2	匹配# 3
库:	n.a.	n.a.	n.a.
组份:	n.a.	n.a.	n.a.
匹配因子:	n.a.	n.a.	n.a.
保留时间:	n.a.	n.a.	n.a.
来源:			
采集日期:	n.a.	n.a.	n.a.
仪器方法:	n.a.	n.a.	n.a.

Chromeleon (c) Dionex
版本 7.2.0.3765
